# Gene expression and locomotor recovery in adult rats with spinal cord injury and plasma-synthesized polypyrrole/iodine application combined with a mixed rehabilitation scheme

**DOI:** 10.3389/fneur.2023.1124245

**Published:** 2023-05-23

**Authors:** Angélica Coyoy-Salgado, Carlos Orozco-Barrios, Stephanie Sánchez-Torres, María Guadalupe Olayo, Guillermo Jesus Cruz, Juan Morales-Corona, Roberto Olayo, Araceli Diaz-Ruiz, Camilo Ríos, Laura Alvarez-Mejia, Rodrigo Mondragón-Lozano, Axayacatl Morales-Guadarrama, Ana Lucía Alonso-García, Omar Fabela-Sánchez, Hermelinda Salgado-Ceballos

**Affiliations:** ^1^Researchers for Mexico CONACyT-Instituto Mexicano del Seguro Social, Medical Research Unit in Neurological Diseases, Specialty Hospital, National Medical Center Siglo XXI, Mexico City, Mexico; ^2^Research Center of the Proyecto CAMINA A.C., Mexico City, Mexico; ^3^Instituto Mexicano del Seguro Social, Medical Research Unit in Neurological Diseases, Specialty Hospital, National Medical Center Siglo XXI, Mexico City, Mexico; ^4^Instituto Nacional de Investigaciones Nucleares, Department of Physics, Axapusco, Mexico; ^5^Department of Physics, Universidad Autónoma Metropolitana Iztapalapa, Mexico City, Mexico; ^6^Instituto Nacional de Neurología y Neurocirugía Manuel Velasco Suárez S.S.A., Department of Neurochemistry, Mexico City, Mexico; ^7^Electrical Engineering Department, Universidad Autónoma Metropolitana Iztapalapa, Mexico City, Mexico; ^8^National Center for Research in Imaging and Medical Instrumentation, Universidad Autónoma Metropolitana Iztapalapa, Mexico City, Mexico; ^9^Instituto Nacional de Ciencias Médicas y Nutrición Salvador Zubirán, Mexico City, Mexico; ^10^Researchers for Mexico CONACyT-Centro de Investigación en Química Aplicada, Department of Chemistry Macromolecules and Nanomaterials, Saltillo, Mexico

**Keywords:** spinal cord injury, gene expression, VEGF, Tubb3, biopolymers, plasma-synthesized polypyrrole/iodine, rehabilitation, functional recovery

## Abstract

**Introduction:**

Spinal cord injury (SCI) can cause paralysis, for which effective therapeutic strategies have not been developed yet. The only accepted strategy for patients is rehabilitation (RB), although this does not allow complete recovery of lost functions, which makes it necessary to combine it with strategies such as plasma-synthesized polypyrrole/iodine (PPy/I), a biopolymer with different physicochemical properties than PPy synthesized by conventional methods. After SCI in rats, PPy/I promotes functional recovery. Therefore, the purpose of this study was to increase the beneficial effects of both strategies and identify which genes activate PPy/I when applied alone or in combination with a mixed scheme of RB by swimming and enriched environment (SW/EE) in rats with SCI.

**Methods:**

Microarray analysis was performed to identify mechanisms of action underlying the effects of PPy/I and PPy/I+SW/EE on motor function recovery as evaluated by the BBB scale.

**Results:**

Results showed robust upregulation by PPy/I in genes related to the developmental process, biogenesis, synapse, and synaptic vesicle trafficking. In addition, PPy/I+SW/EE increased the expression of genes related to proliferation, biogenesis, cell development, morphogenesis, cell differentiation, neurogenesis, neuron development, and synapse formation processes. Immunofluorescence analysis showed the expression of β-III tubulin in all groups, a decreased expression of caspase-3 in the PPy/I group and GFAP in the PPy/I+SW/EE group (*p* < 0.05). Better preservation of nerve tissue was observed in PPy/I and PPy/SW/EE groups (*p* < 0.05). In the BBB scale, the control group scored 1.72 ± 0.41, animals with PPy/I treatment scored 4.23 ± 0.33, and those with PPy/I+SW/EE scored 9.13 ± 0.43 1 month after follow-up.

**Conclusion:**

Thus, PPy/I+SW/EE could represent a therapeutic alternative for motor function recovery after SCI.

## 1. Introduction

Spinal cord injury (SCI) can cause permanent sensory and motor dysfunctions and paralysis (1, 2). In addition to the functional deficits that patients with an SCI have for life, they will also have to deal with a huge financial burden, which will impact their families, society, and health systems. This happens because therapeutic strategies for complete functional recovery have not yet been developed. Currently, rehabilitation (RB) is the only therapeutic strategy accepted worldwide for the treatment of these patients. Although RB benefits are limited and it does not allow the recovery of all lost functions after an SCI, it has been shown that RB in its different schemes modifies the expression of molecules related to regeneration and plasticity (3–7).

Several studies indicate that RB by swimming (SW) applied after SCI maintains and strengthens synaptic connections, promotes the expression of brain-derived neurotrophic factor (BDNF) and neurotrophin-3 (NT-3) (8), and improves motor function recovery (9–11).

Another kind of RB is provided by an enriched environment (EE), which consists of different elements that allow voluntary and free physical activity individually or in groups (12). RB by EE has been used in the treatment of different neurological diseases and has been shown to promote the expression of genes and molecules involved in the processes of neurogenesis, regeneration, and plasticity (13–15). Nevertheless, RB benefits are limited, and it does not allow the recovery of all lost functions (16).

In previous studies, our research group demonstrated the beneficial effect of using plasma-synthesized polypyrrole/iodine (PPy/I) after SCI in rats. PPy/I decreases inflammatory response, neuroprotects spinal cord tissue, reduces glial scar formation, and promotes the expression of molecules related to nerve plasticity and regeneration, as well as the recovery of motor function (17–21). In addition, our research group recently demonstrated that the combination of plasma-synthesized PPy/I and a mixed scheme of rehabilitation by SW/EE promotes nerve tissue preservation and the expression of nerve plasticity-related molecules 2 months after SCI (19).

Thus, in the present study, following SCI with or without the application of PPy/I or PPy/I and SW/EE, gene expression was analyzed by microarray analysis, identifying genes that could explain some possible mechanisms involved in motor function recovery in experimental animals. Although gene expression as the global epigenetic mechanism underlying these effects remains unknown, the increase in gene expression reported in the present study and its relationship with certain functional effects observed will allow for progress in this area of knowledge.

## 2. Materials and methods

### 2.1. Synthesis of the polymer PPy/I

**Figure 1:**
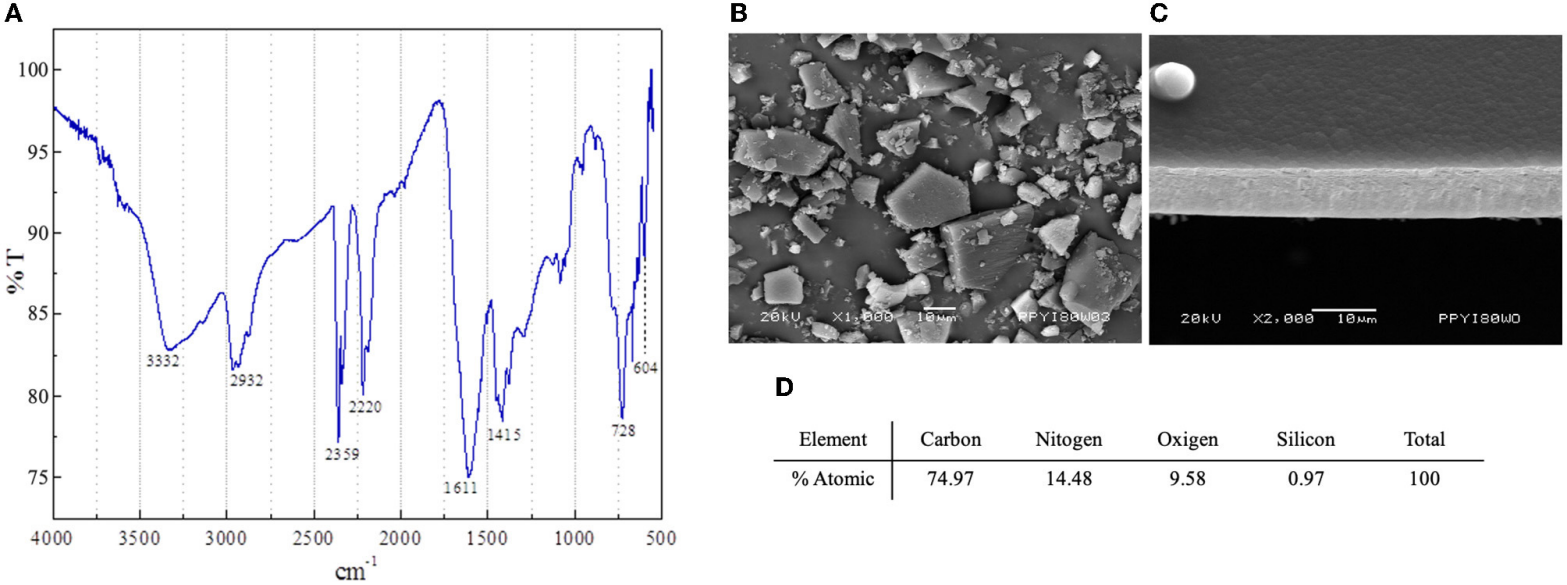
Characterization of polypyrrole-Iodine (PPy/I) synthesized by plasma. Fourier Transform Infrared (FT-IR) transmittance spectrum **(A)**; Scanning electron microscopy (SEM) photomicrograph of PPy/I particles obtained after grinding a thin film **(B)**; SEM photomicrograph of PPy/I thin film before grinding **(C)**; Elemental atomic % of carbon, oxygen and nitrogen in PPy/I **(D)**.

The polymer PPy/I was synthesized and characterized as described previously (18, 20–22). Briefly, the polymer was synthesized in a tubular glass reactor of 25 cm. The pyrrole monomer (Sigma-Aldrich, St. Louis, MO, USA, 98%) and dopant iodine (Aldrich, 99.8%) were placed in separate containers and connected to the reactor by the lateral access ports. The process was started by reducing the pressure inside the reactor to 10^−2^ mbar, and then electric shocks were produced in the air with an Advanced Energy RFX-600 power generator set to 13.5 MHz, 700 V, and 80 W for 240 min (18, 20–22). This process generates thin films of PPy/I that adhered to the internal walls of the reactor. The films were swollen with acetone, washed with distilled water, removed from the walls, dried, and manually ground in an agate mortar for 10 min to obtain particles of different sizes and geometries (mesoparticles). The characterization of the plasma-synthesized PPy/I was performed by an infrared (IR) spectroscopy on a Thermo Scientific Nicolette iS5 spectrophotometer using 32 scans with a wavelength range of 400–4,000 cm^−1^ and a resolution of 4 cm-1. The morphological analysis was performed with a Jeol IT-100 scanning electron microscope using an acceleration voltage of 20 kV. The elemental analysis was done in a microscope that was coupled with an EDS Oxford INCA-XACT energy dispersion probe (Figure 1). The images were processed with the Olympus Measure IT program (21).

Once the materials were characterized, 5 mg of PPy/I mesoparticles were added to 1 ml of saline solution (physiological solution, 0.9% sodium chloride), placed in an ultrasonic agitator for 30 min to disintegrate the agglomerates, and sterilized in an autoclave (17, 18, 20, 21) to place the polymer at the site of injury in the spinal cord of the animals from the corresponding groups.

### 2.2. Animals

Adult female Long-Evans rats with 250–300 g of body weight were housed under standard conditions (12-h light/dark cycles, 22°C). Only female rats were included because although several studies in rodents with SCI have confirmed a protective bias for the female sex (23, 24), others have not found any difference (25, 26). Moreover, SCI prevents animals from lifting the back of the body and therefore dragging it to move, which in the case of male rats, causes lesions and infections in the scrotum that may compromise their state of health. All surgical and experimental procedures were carried out in accordance with the Regulation of the Mexican General Law of Health regarding research and science (27) and the Mexican Guidelines for Animal Care and Handling (NOM-062-ZOO-1999) (28) with the authorization of the National Committee for Scientific Research of the Mexican Institute of Social Security. All efforts were made to minimize animal discomfort and reduce the number of animals used.

Three groups were randomly formed: an untreated group (SCI), the group that received PPy/I, and the group treated with PPy/I+SW/EE.

### 2.3. Surgical procedure

The animals were anesthetized intramuscularly with a mixture of ketamine and xylazine at 75 and 25 mg/kg of body weight, respectively. Laminectomy was performed at the thoracic 9 vertebra (T9) level, and then an SCI was induced by using the NYU impactor stereotactic (29, 30). The presence of a hematoma at the lesion site was verified under a microscope, and then the muscle and skin were sutured in layers. Subsequently, an antibiotic (benzathine penicillin) was administered intramuscularly as a single dose (1,200,000 IU) and an analgesic on the water to drink (paracetamol, 5 ml/l of water) for 5 days. The animals were placed in individual boxes in the vivarium under the conditions previously described. The neurogenic bladder and the intestine were manually emptied every day until the animal regained sphincter control. The surgical wound was checked daily, and the general health of each animal was verified.

### 2.4. Treatments

PPy/I: 48 h after SCI, 5 mg/ml of PPy/I were administered by injecting a total volume of 30 μl directly into the spinal cord site of injury, while the animal was under anesthesia (17).

Rehabilitation: RB started 15 days after SCI in order to avoid increasing spinal cord damage due to the susceptibility of the nervous tissue during the initial stages after an injury of this nature (9, 31, 32).

Pre-training: The animals from the group that received SW/EE were subjected to a period of adaptation to the new environment 1 week before the induction of SCI. Each day, they were placed in a glass tank (149 × 18.5 × 30 cm) filled with water at a controlled temperature and with a non-slip acrylic platform at the end of the lane, so that the animal could rest during SW sessions. The SW sessions initially had 2 min of duration with increments of 1 min per session until 6 min that was reached at the end of the pre-training week (9). The animals received seeds or cereals as a reward at the end of the activity.

RB by Swimming (SW): The animals were placed in the tank with warm water every third day, with 2 min sessions that increase by 1 min each week until the end of 4 weeks (33). In each session, the animals received support from the rehabilitator to carry out the activity while regaining control of the posterior train. The hind limbs were massaged to relax the muscles and prevent atrophy (34) and passive-assisted mobilizations with circular movements, as allowed by each articulation were performed to facilitate the relearning of movement and control during SW.

RB in Enriched Environment (EE): The animals from the corresponding groups were placed in an EE area containing articles of different colors and textures, including exercise wheels, plastic tubes for labyrinths, exercise bars, and obstacle circuits, during 2 h daily until the end of the study. The location of the items and their distribution were modified periodically to avoid the habituation of the animals to the EE and to increase motor-sensory stimulation through novel activities. Social interaction with 5 or 6 other rats was also facilitated at the same time as the exploration of the environment (35).

### 2.5. Evaluation of functional recovery

One day after the surgical procedure, the animals were verified to have complete paralysis of the posterior extremities and, a week later, the weekly evaluation of motor recovery was performed using the Basso, Beattie, and Bresnahan functional scale (BBB) (36, 37). The BBB evaluates the movement and range of motion in all three hind limbs joints: hip, knee, and ankle, and whether the animal can support its body weight and coordinate movement between the back and the anterior extremities should also be evaluated. According to the degree of recovery of motor function that the animal achieves or does not achieve during 4 weeks of evaluation, it is given a score ranging from 0 to 21, where 0 indicates no movement and 21 represents a normal gait.

### 2.6. Tissue collection

The animals included in the study were euthanized 1 month after SCI, according to the Official Mexican Standard regarding the humane euthanasia of animals (NOM-033-SAG/ZOO-2014 and NOM-033-ZOO1995) (38, 39). Animals for immunofluorescence study were anesthetized with pentobarbital and perfused intracardially with 200 ml of saline solution plus 1000UI of heparin at room temperature, followed by 250 ml of 4% paraformaldehyde and glutaraldehyde (1:1,000). After perfusion, the spinal cord, including the epicenter of the lesion plus 1 cm in the rostral and 1 cm in the caudal directions, was extracted. The tissues were dehydrated, embedded in paraffin, and sectioned longitudinally at 6 microns thick from the periphery to the epicenter in a Leica microtome. Two slides, each containing 5 sections, were selected for each animal, with the ependymal canal used as a reference. Euthanasia of the animals for gene expression microarray assay was performed by decapitation using a guillotine designed for small animals (World Precision Instruments, Inc. Sarasota, FL USA; Model DCAP-M, serial 133708 9K) under optimal conditions of use. Euthanasia was performed by specialized personnel in a room where only one animal was placed at a time. The animal was delicately taken from its cage using a fleshy glove and its head was placed in the guillotine proceeding to the decapitation in a single movement. Subsequently, the spinal cord, including the epicenter of the lesion plus 0.5 cm in the rostral direction and 0.5 cm in the caudal direction, was collected fresh at 4°C and immediately stored at −70°C until RNA extraction. The body of the animal was removed from the room, placed in a yellow bag, and taken to the corresponding area of the bioterium and, the area used was cleaned before introducing the next animal.

Total RNA was extracted from spinal cords using TRIzol Reagent (Invitrogen) according to the manufacturer's instructions. RNA integrity was assessed using the Agilent 2100 Bioanalyzer (Agilent Technologies, Santa Clara, CA, USA) and the software 2100 Expert. Only, the samples with an RNA integrity number (RIN) value above 8.0 were used for further processing.

### 2.7. Gene expression microarray assay

Microarray experiments were performed in the Microarray Core Facility at the National Institute of Genomic Medicine (INMEGEN, Mexico City, Mexico). Target cDNA was prepared according to the Whole-Transcript Sense Target Labeling Protocol (P/N 703174, Thermo Fisher Scientific). The labeled cDNA product was hybridized to the GeneChip rat Clariom S microarray (Affymetrix). The samples were washed with low-stringency and high-stringency buffers and were stained with streptavidin–phycoerythrin using the GeneChip Fluidics Station 450 with the FS450_0002 protocol. The GeneChip Scanner 3000 7G (Affymetrix) was used to collect fluorescent signals, and Expression Console software (Affymetrix) was used to obtain intensity signal and quality data of the scanned arrays. Raw microarray intensity data were pre-processed and quantile normalized using the Transcriptomic Analysis Console (TAC) Software 4.0.1 (Thermo Fisher Scientific).

An unpaired one-way ANOVA and a false discovery rate (FDR) analysis were performed. Results obtained with the TAC Software 4.0.1 were reported. Using a detection technique of the more than 20,000 transcripts represented on the Affymetrix rat GeneChip, only those transcripts that showed a differential gene expression with a fold change (Fc) >2 and *p* < 0.05 were considered statistically significant and non-random and were analyzed in the present study. The original data were pre-processed by the RMA function with the Affymetrix package of R language (40). The original CEL files were switched into probe expression measures, and the probe-level data were converted into gene names by an annotation package supported by the GCBI platform. After the normalization and statistical analysis of the raw microarray data, all treatments were compared against the SCI untreated group.

Validation of Selected Differentially Expressed Genes by RT-qPCR: The genes with the highest Fc among treatment comparisons and with relevance in the enriched biological processes and cellular component were selected. Thus, Tubb3, Tubb4, and VEGFb were selected for the gene expression validation, where PPy/I increased the expression of the Tubb3 gene, while PPy/I+SW/EE increased the expression of Tubb 4 and VEGFb.

cDNA was synthesized from 1 μg of total RNA using the M-MVL reverse transcriptase according to the manufacturer's instructions (Invitrogen, Carlsbad, CA, USA) with oligo-dT12–18 as primers. The reaction was placed in a thermocycler (Bio-Rad, USA) using the following program: 10 min at 25°C for one cycle, 50 min at 37°C for one cycle, and 15 min at 70°C for one cycle.

The qPCR assay was performed with UPL probes for vascular endothelial growth factor B (Vegfb) NM_053549.1 (cat. no. 04688651001), tubulin β class IVa (Tubb4) NM_080882.1 (cat. no. 04687965001), and tubulin β class III (Tubb3) NM_139254.2 (cat. no. 04686942001) (Roche, Germany). Transcripts of the Hprt1 gene, own design, were used as a housekeeping gene. The following TaqMan primers and probes were used: Hprt1 Fw: 5'-CAG ACT TTG GAT TTG AA-3', Probe: 5'-CCA GAC AAG TTT GTT GGA TAT GCC CTT-3', Rv: 5'-ATC CCT GAA GTG CTC ATT ATA G-3' (PrimeTime^®^ qPCR Assays, IDT DNA, USA).

The Vegfβ, Tubb4, Tubb3, and Hprt1 genes of samples were amplified with the LightCycler TaqMan Master Mix reagent (Roche, Germany) and detected on a Light Cycler 2.0 thermal cycler (Roche, Germany). Relative quantification was assessed using the formula 2-ΔΔCT and by normalizing the amount of the target gene to the Hprt1 housekeeping gene.

All data were analyzed and plotted using the GraphPad Prism 5.0 software for Windows XP (GraphPad Software, CA, USA). Statistical analysis was performed using one-way ANOVA followed by Bonferroni *post hoc* test for the gene expression experiment. Values of *p* < 0.05 were considered statistically significant.

### 2.8. Morphometric analysis

One slide, which contains five sections, was selected for each animal and stained with hematoxylin-eosin. Panoramic images were taken with a Leica clear field microscope. Quantification of the preserved tissue was done by using FIJI software. The total area of each section was determined, and the thinning of the spinal cord and the presence of microcysts and cysts were evaluated.

### 2.9. Immunofluorescence analysis

The selected slides were placed in an oven at 60°C to remove excess paraffin, dehydrated in a series of graded alcohols, and placed in a Coplin jar with 10 mM citrate buffer at pH 6, inside a pressure cooker for 20 min to perform antigen recovery. Then, the tissues were permeabilized with PBS-T (0.01 M PBS and 0.1% Triton) for 30 min. Non-specific sites were blocked with 5% horse serum in PBS-T in a humid chamber for 30 min, and the tissues were incubated with goat anti-GFAP (1:500, Abcam), rabbit anti-cleave caspase-3 (1:300, Cell Signaling), and mouse anti–βIII-tubulin (1:500, Abcam) primary antibodies in a humid chamber at 4°C overnight. Afterward, the tissues were washed with PBS-T and incubated with Alexa Fluor^®^ 488 donkey anti-rabbit IgG (1:500, Molecular Probes), Alexa Fluor^®^ 594 donkey anti-goat IgG (1:500, Molecular Probes), and Alexa Fluor^®^ 405 donkey anti-mouse IgG (1:500, Molecular Probes) secondary antibodies for 2 h at room temperature in the dark. Then slides were placed in a solution of 0.1% Black Sudan B (Sigma-Aldrich) in 70% ethanol for 15 min, the excess Black Sudan B was removed using distilled water and PBS-T. The tissues were covered with VECTASHIELD^®^ and a coverslip and were observed under a confocal Nikon TI eclipse microscope.

## 3. Results

### 3.1. Gene expression

**Figure 2:**
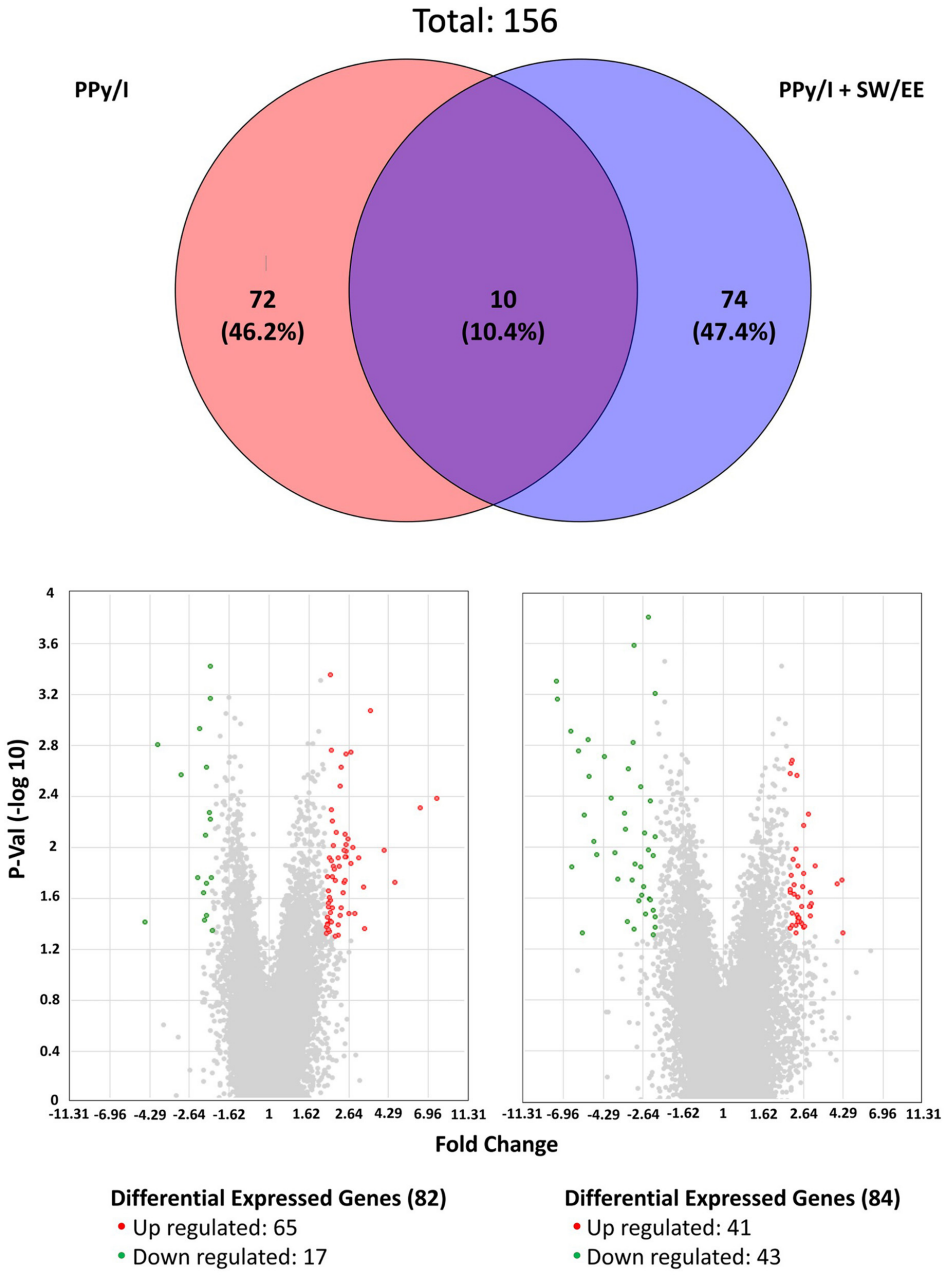
Plasma-synthesized polypyrrole/iodine (PPy/I) and PPy/I plus swimming and enriched environment (PPy/I + SW/EE), treated groups promote changes in the gene expression profile of spinal cord injury (SCI). Venn's diagram was constructed by using the genes that changed their expression with the application of PPy/I and PPy/I + SW/EE treatments when compared with the untreated spinal cord injury group. PPy/I differentially regulated 82 genes while PPy/I + SW/EE regulated the expression of 84 transcripts. Volcano plot of differentially expressed genes (DEGs) between spinal cord injury (SCI) group without treatment and PPy/I or PPy/I + SW/EE treated groups. Volcano plot of DEGs screened on the basis of |logFC| > 2 and adjusted *p*-value < 0.05. The red points represent up-regulated genes, the green points represent down-regulated genes, and the gray points represent genes with no significant difference.

Gene expression microarrays were used to identify differentially expressed genes (DEGs). Using an adjusted *p*-value of < 0.05 and |logFC| > 2 as the threshold, a volcano plot developed with R software showed the distribution of all DEGs. The results obtained are included as Supplementary material and showed that PPy/I modified the expression of 82 genes, of which 65 increased their expression and 17 decreased it (Supplementary Table 1), while PPy/I+SW/EE modified the expression of 84 genes, of which 41 increased their expression and 43 decreased it (Supplementary Table 2 and Figure 2).

Enrichment analysis of gene ontology categories using the database Protein ANalysis THrough Evolutionary Relationships (PANTHER) was performed to identify possible biological processes amended by PPy/I or by PPy/I+SW/EE 1 month after SCI. The lists of the DEGs are included in the supplementary material (Supplementary Tables 3, 4), which shows genes related to “molecular function,” “biological processes,” “cellular component,” and “protein class” that were classified under one or more Gene Ontology (GO) categories. Cross-examination using the DAVID enrichment algorithm (41, 42) was performed (Supplementary Tables 3, 4).

**Figure 3:**
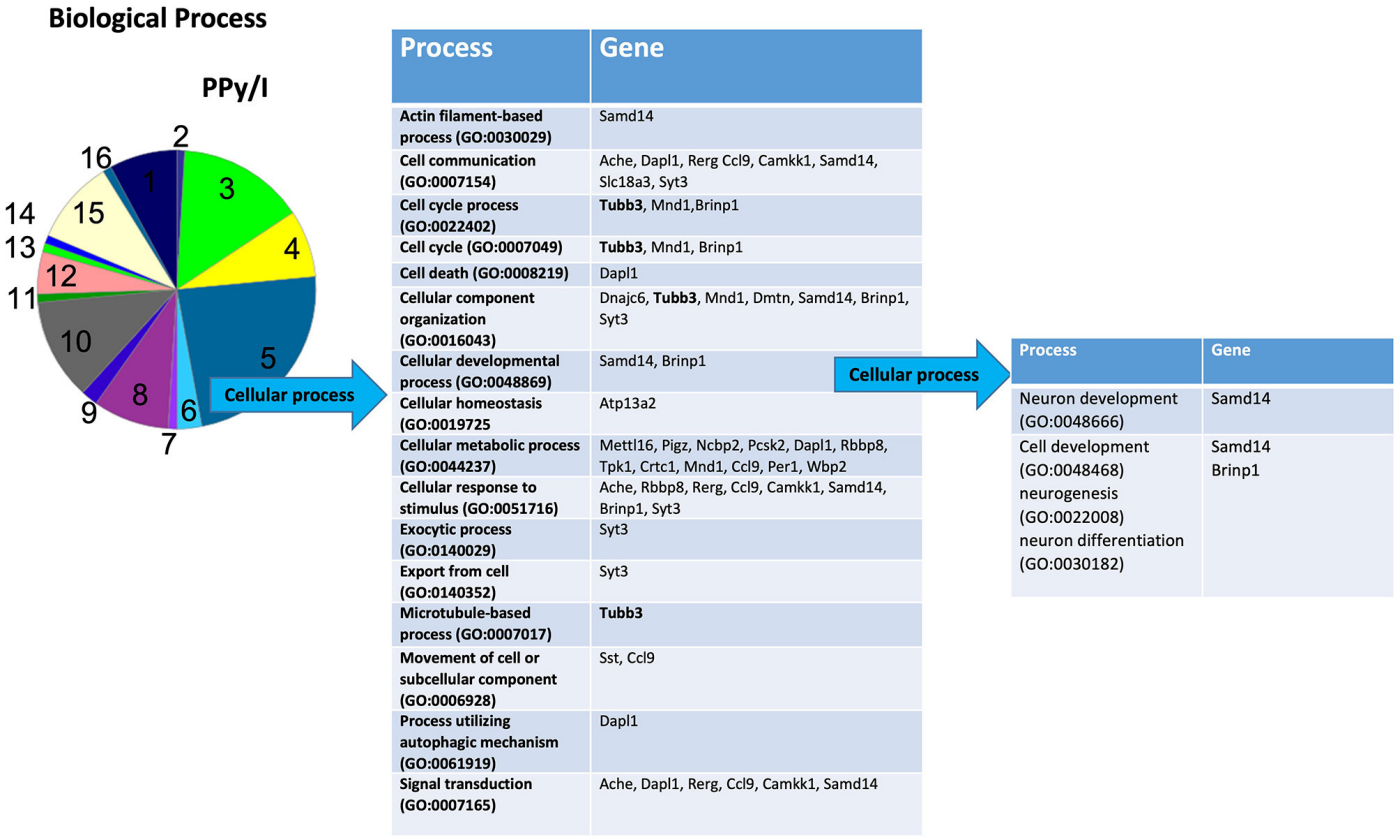
Enriched analysis of the genes group regulated by plasma-synthesized polypyrrole/iodine (PPy/I) application. The proteins coded by the differentially expressed genes after PPy/I treatment were analyzed with PANTHER. The diverse biological processes in which the gene products participate are shown. As the category of cellular process was the most enriched by PPy/I application, a second analysis was performed with the aim to determine sub-categories. The pie charts show the enriched categories for PPy/I treatment after a spinal cord injury (SCI).

**Figure 4:**
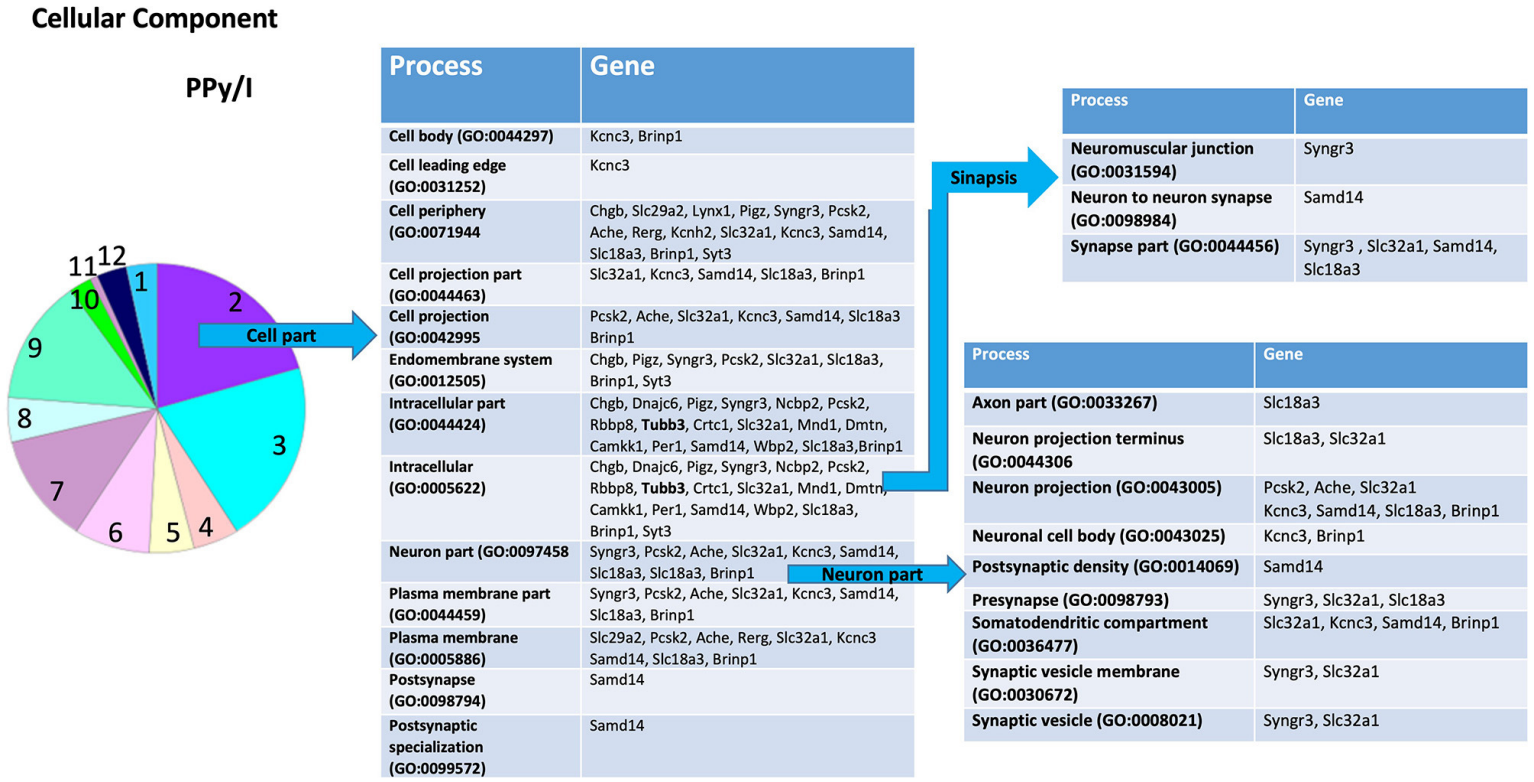
Enrichmed analysis of the group of genes regulated by plasma-synthesized polypyrrole/iodine (PPy/I) application. The proteins coded by the genes differentially expressed after PPy/I treatment were analyzed with PANTHER. The diverse cellular processes in which the gene products participate are shown in the tables. As the category of cell part was the most enriched by PPy/I, a second analysis was performed to determine sub-categories. The pie charts show the enriched categories for PPy/I treatment after a spinal cord injury (SCI).

**Figure 5:**
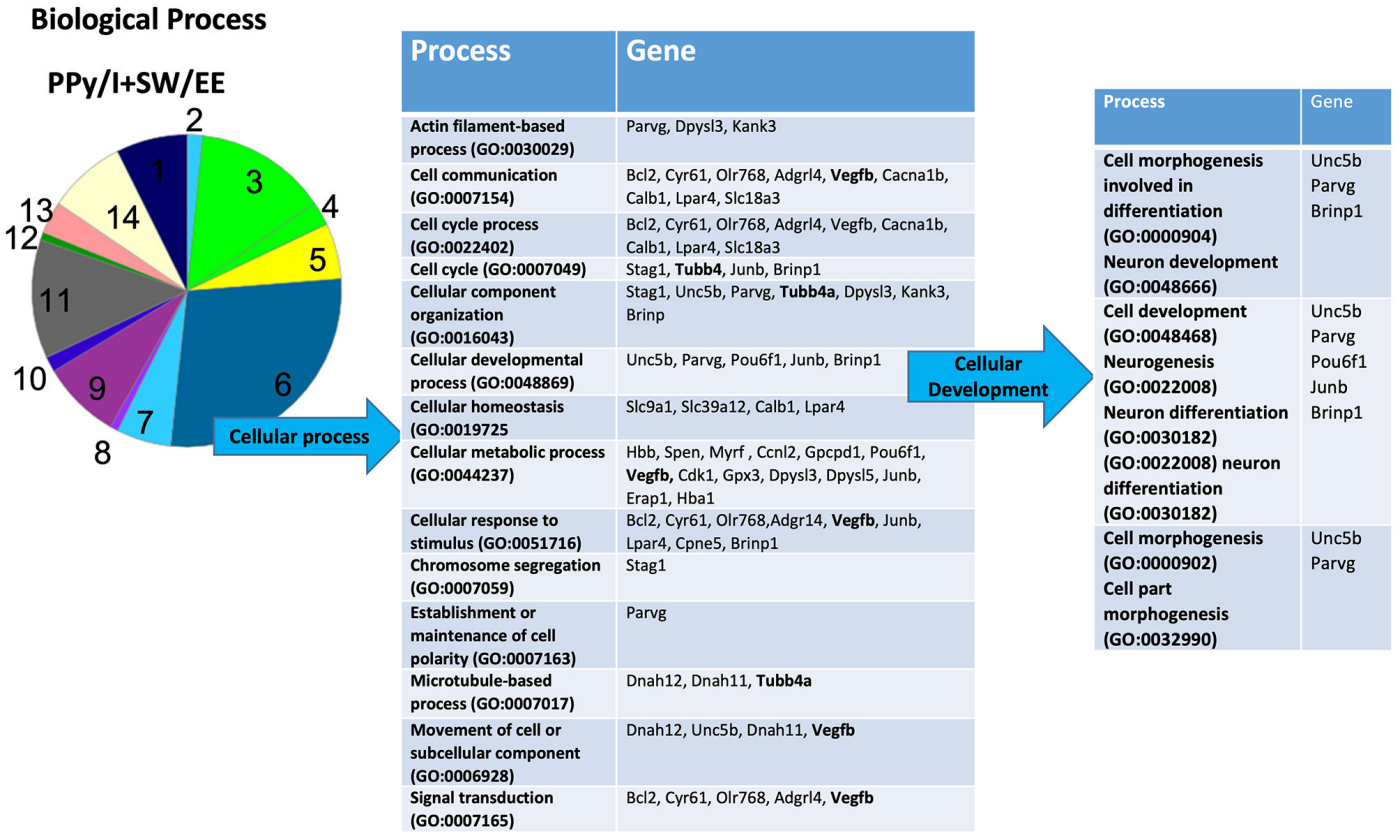
Enrichment analysis of the group of genes regulated by plasma-synthesized polypyrrole/iodine and a swimming and enriched environment (PPy/I + SW/EE). The proteins coded by the differentially expressed genes after PPy/I + SW/EE treatment were analyzed with PANTHER. The diverse biological processes in which the products of the participating gene are shown. As the category of cellular processes was the most enriched by PPy/I, we performed a second analysis to determine sub-categories. The pie charts show the enriched categories for PPy/I +SW/EE treatment after a spinal cord injury (SCI).

**Figure 6:**
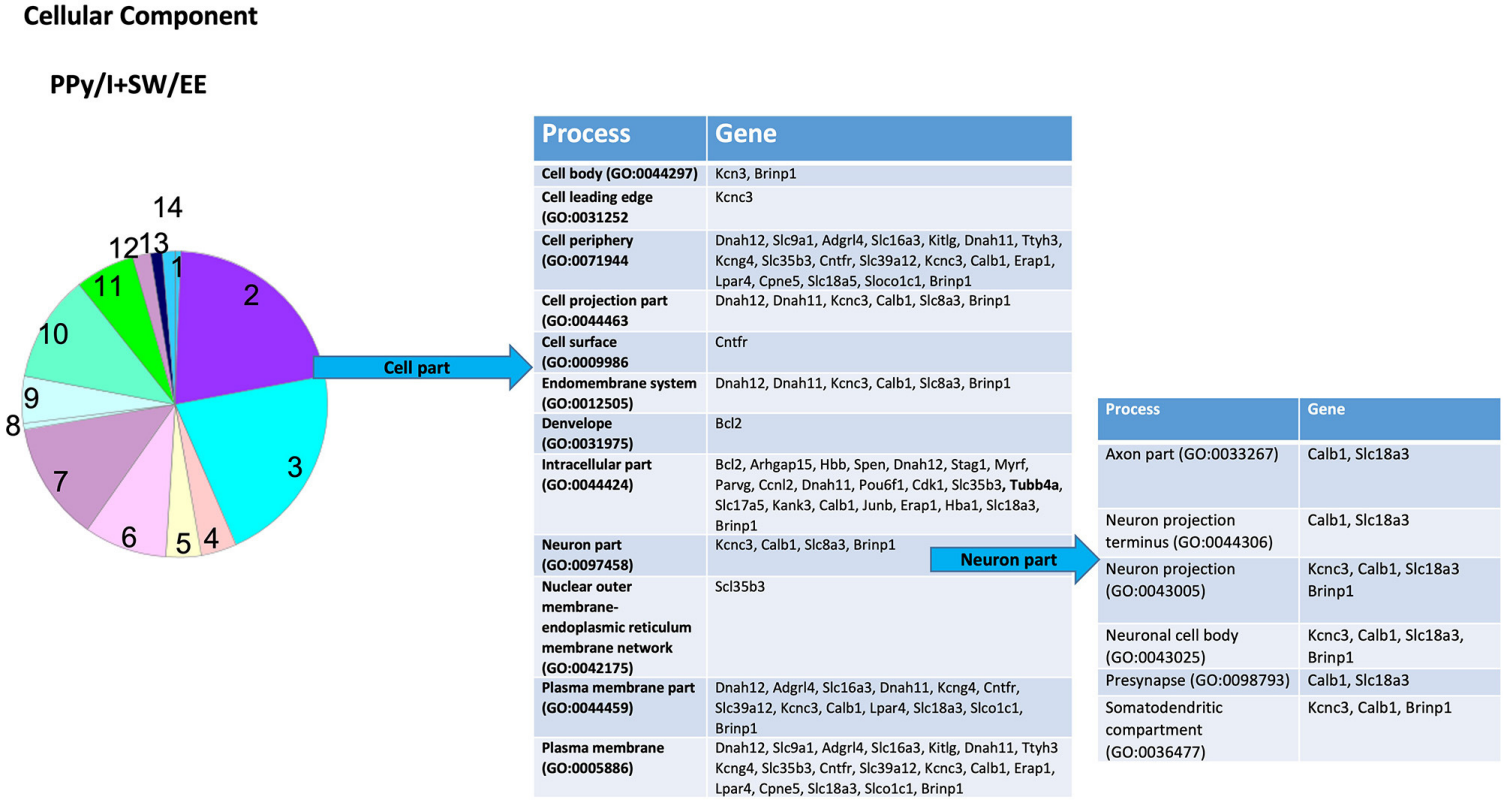
Enrichment analysis of the group of genes regulated by plasma-synthesized polypyrrole/iodine (PPy/I) plus swimming and enriched environment treatments (PPy/I + SW/EE). The proteins coded by the differentially expressed genes after PPy/I + SW/EE treatment were analyzed with PANTHER. The diverse cellular processes in which the gene products participate are shown in the tables. As the category of cell part was the most enriched by PPy/I + SW/EE, a second analysis was performed to determine sub-categories. The pie charts show the enriched categories for PPy/I + SW/EE treatment after a spinal cord injury (SCI).

In the PANTHER database, the most enriched categories in “biological processes” were “cellular process,” while in the category “cellular components” were “cell part” for PPy/I (Figures 3, 4) and PPy/I+SW/EE (Figures 5, 6).

According to the PANTHER database, PPy/I administration modified some genes in the sub-category “cellular process,” a derivate of the “biological processes” category. The sub-category “cellular process” includes processes such as “actin filament-based process,” “cell communication,” “cell cycle,” “cell death,” “cellular component organization,” “cellular development,” “cellular homeostasis,” “cellular metabolic process,” “cellular response to stimulus,” “exocytic process,” “export from cell,” “microtubule-based process,” “movement of cell or subcellular component,” “process utilizing autophagic mechanism,” and “signal transduction” (Figure 3).

In the sub-category “cellular developmental,” PPy/I-regulated genes include processes such as “neurogenesis,” “neuronal development,” and “neuronal differentiation” (Figure 3). In the sub-category “cell part,” a derivate of “cellular component” category, the sub-category “cell part” includes processes such as “cell body,” “cell leading edge,” “cell periphery,” “cell projection part,” “cell projection,” “endomembrane system,” “intracellular part,” “neuron part,” “plasma membrane part,” “postsynapse,” and “postsynaptic specialization.” The sub-category “neuron part” contains genes related to processes such as “axon part,” “neuron projection,” “neuronal cell body,” “postsynaptic density,” “presynapse,” “somatodentritic compartment,” and “synaptic vesicle membrane” (Figure 4).

According to the PANTHER database, the combined therapy with PPy/I+SW/EE modified some genes in the sub-category “cellular processes,” a derivate of the “biological processes” category. The sub-category “cellular processes” includes processes such as “actin filament-based process,” “cell communication,” “cell cycle,” “cellular component organization,” “cell development,” “cellular homeostasis,” “cellular metabolic process,” “cellular response to stimulus,” “chromosome segregation,” “establishment or maintenance of cell polarity,” “microtubule-based process,” “movement of cell or subcellular component,” and “signal transduction” (Figure 5), whereas the sub-category “cellular development” includes processes such as “neurogenesis,” “differentiation,” and “cellular morphogenesis” (Figure 5).

The PPy/I+SW/EE treatment-modified genes are included in the sub-category “cell part,” a derivate of the “cellular component” category. The sub-category “cell part” includes processes such as “cell body,” “cell leading edge,” “cell periphery,” “cell projection part,” “cell projection,” “cell surface,” “endomembrane system,” “development,” “intracellular part,” “neuron part,” “nuclear outer membrane-endoplasmic reticulum membrane network,” and “plasma membrane part.” The sub-category “neuron part,” contains genes related to processes such as “axon part,” “neuron projection,” “neuronal cell body,” “presynapse,” and “somatodentritic compartment” (Figure 6).

In the DAVID database, the most functional annotation clustering to PPy/I with an enrichment score of 1.43 included methylation (eight genes), synapse (five genes), cell junction (five genes), and cell membrane (six genes). In PPy/I+SW/EE, combined therapy with an enrichment score of 5.48 included hemoglobin complex (seven genes), oxygen transport (seven genes), globin (seven genes), globin-like (seven genes), globin structural domain (seven genes), oxygen transport (seven genes), oxygen transporter activity (seven genes), metal ion-binding site: iron (heme distal ligand) (six genes), oxygen binding (seven genes), metal ion-binding site: iron (heme proximal ligand) (six genes), African trypanosomiasis (seven genes), malaria (seven genes), hemoglobin, beta (four genes), heme (seven genes), and hydrogen peroxide catabolic process (four genes), haptoglobin binding (three genes), heme binding (seven genes), iron (eight genes), haptoglobin–hemoglobin complex (three genes), hemoglobin, pi (three genes), chain: hemoglobin subunit beta-2 (three genes), iron ion binding (seven genes), hemoglobin, alpha (three genes), response to hydrogen peroxide (five genes), sequence variant (six genes), positive regulation of cell death (four genes), peroxidase activity (three genes), erythrocyte development (three genes), protein heterooligomerization (four genes), blood microparticle (four genes), S-nitrosylation (three genes), polymorphism (three genes), metal-binding (11 genes), and acetylation (eight genes) (Supplementary Tables 5, 6).

In general, some of the PPy/I-regulated genes were involved in the development process, biogenesis, synapses, and synaptic vesicle traffic, while PPy/I+SW/EE-modified gene expression was involved in proliferation, biogenesis, cell development, neuron development, morphogenesis, cell differentiation, neurogenesis, and synapse.

For gene expression validation, the treatments used for the microarray analysis were included. PPy/I modified the expression of the Tubb3 gene, while PPy/I+SW/EE-modified Tubb 4 and VEGFβ promote the expression of molecules involved in the neurogenesis, regeneration, and plasticity processes (13). Thus, these genes were selected to validate the microarray.

**Figure 7:**
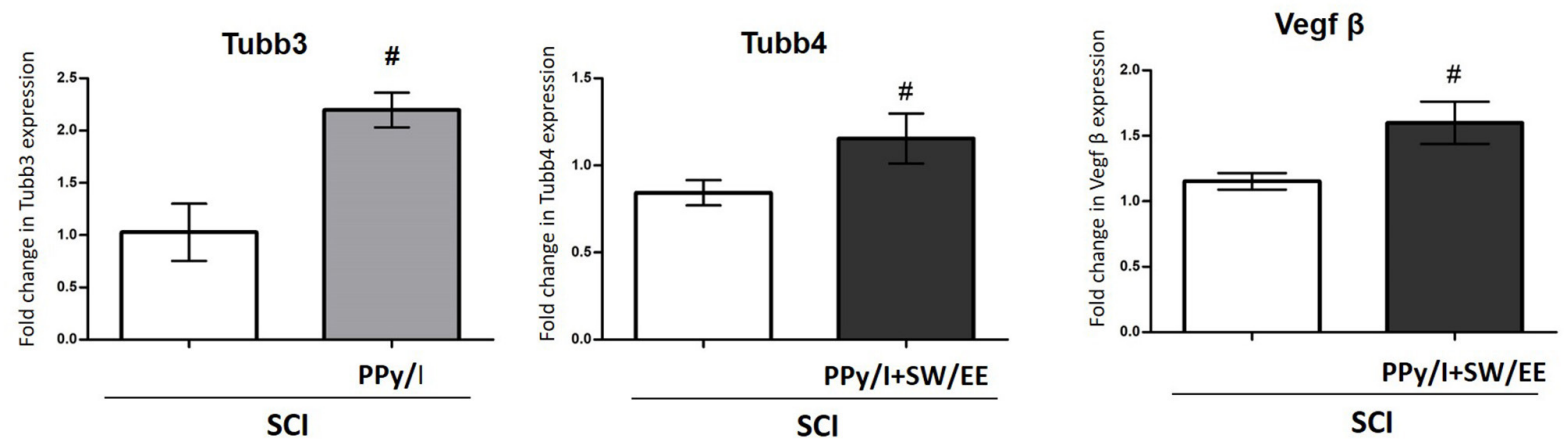
Relative gene expression of Tubb3, Tubb4 and vascular endothelial growth factor (VEGFβ) after spinal cord injury (SCI) without treatment or after plasma-synthesized polypyrrole/iodine (PPy/I) or PPy/I plus swimming and enriched environment (PPy/I + SW/EE) treatment. RNA extraction followed by RT-qPCR was carried out in the spinal cord after a SCI in rats treated with PPy/I or PPy/I + SW/EE. Relative expression of Tubb3, Tubb4 and VEGFβ was normalized to Hprt1 expression. Each bar represents the mean ± SD (*n* = 5). A Student's *t*-test analysis was performed. ^#^*p* < 0.05 vs. SCI.

The microarray experiments showed that the expression of Tubb3 (Fc 2.03) increased in the group treated with PPy/I, while Tubb 4 (Fc 2.18) and VEGFβ (Fc 2.04) increased in the group treated with PPy/I+SW/EE. Remarkably, the RT-qPCR data indicated that PPy/I increased the expression of the Tubb3 gene (2.196 ± 0.16), and PPy/I+SW/EE regulated the expression of Tubb4 (1.12 ± 0.20) and VEGFβ (1.59 +0.16) genes (Figure 7).

### 3.2. Motor functional recovery

**Figure 8:**
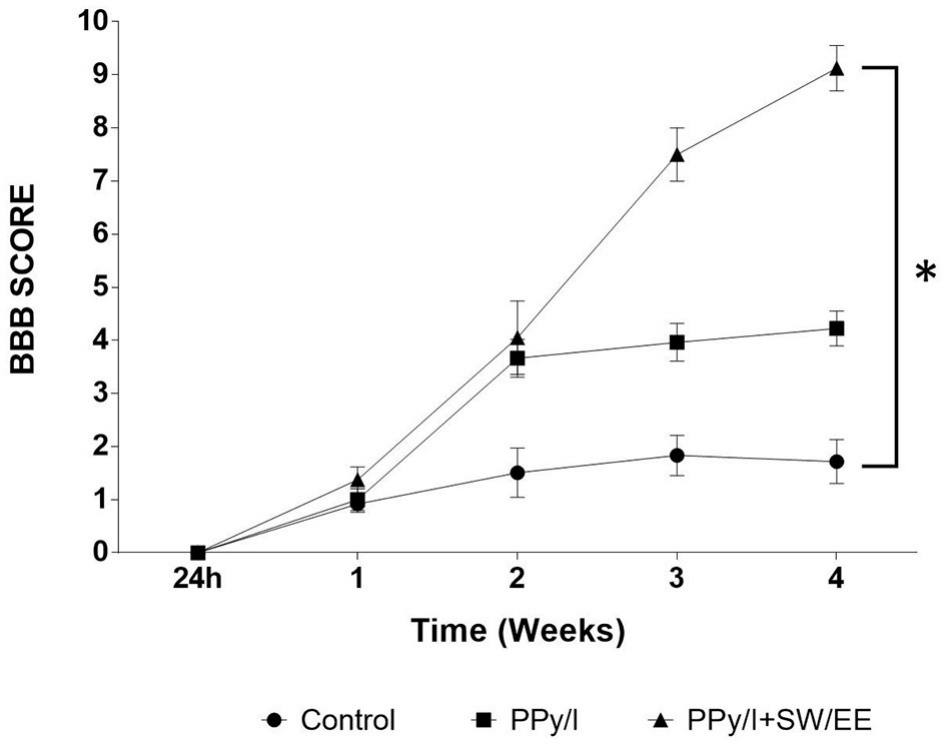
Recovery of locomotor function, as evaluated in an open field with the Basso, Beathie and Bresnaham scale (BBB). The results are expressed as the mean ± SEM, plasma-synthesized polypyrrole/iodine (PPy/I) and combined treatment of PPy// with swimming and enriched environment (PPy/I+SW/EE). Repeated measures ANOVA followed by a Tukey *post hoc* test *(*p* < 0.0001).

Motor functional recovery was evaluated according to the BBB scale (Figure 8), where the control group obtained a final average score of 1.72 ± 0.41, which means that most of the animals exhibited slight movement of one of the two joins, usually the hip. Animals treated with PPy/I were able to have mild hip, knee, and ankle movements consistently from the second week of evaluation, obtaining a score of 4.23 ± 0.33 in the fourth week, while the animals that received the combined strategy of PPy/I+SW/EE scored 9.13 ± 0.43, which implies that these animals showed wide hip, knee, and ankle movements and plantar placement of the paw with consistent weight support (*p* < 0.05).

The combination of PPy/I and the mixed RB scheme (SW/EE) allows the animals to show a faster motor recovery compared to that achieved with the administration of PPy/I alone. The animals in the PPy/I+SW/EE group showed slight movements in the hip, knee, and ankle from the second week of evaluation of functional recovery when RB therapies were initiated.

### 3.3. Morphometric analysis

**Figure 9:**
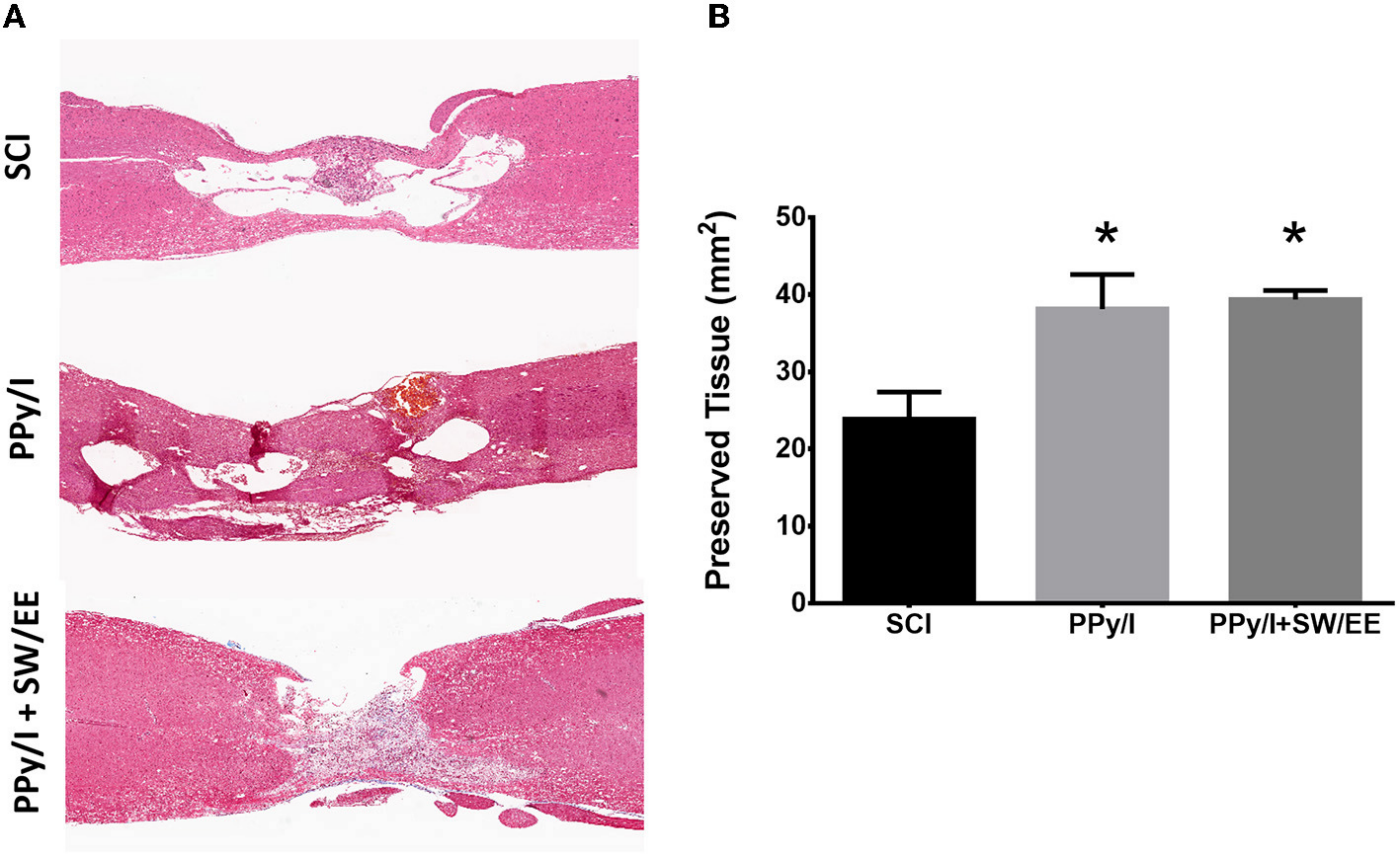
Histological analysis by staining with haematoxylin-eosin. **(A)** The epicenter of the injury of the animals that received PPy/I or PPy/+SW/EE was mostly occupied by tissue, while in the control group, a cystic zone surrounded by microcysts was formed and vacuolar structures containing fragments of degenerating axons and cellular debris were identified. In the group that received PPy/I+SW/EE treatment, the presence of neurons was observed in the rostral and caudal regions, while in the group that received only PPy/I treatment, some neurons in good conditions and others that were in the process of cell death were observed in the rostral zone of the spinal cord. **(B)** Morphometric analysis showed better preservation tissue in animals that received PPy/I or PPy/+SW/EE treatments. One-way ANOVA followed by a Tukey *post hoc* test *p < 0.05.

After 1 month of follow-up, histological analysis was performed by using hematoxylin-eosin staining to identify changes in the cytoarchitecture of the rostral and caudal areas of the spinal cord as well as in the epicenter of the lesion of animals from each experimental group. The epicenter of the injured spinal cord in animals receiving PPy/I or PPy/+SW/EE was occupied primarily by tissue, while in the control group, a cystic zone was formed and around the site of injury, vacuolar structures containing fragments of degenerating axons and cellular debris were identified. Numerous inflammatory cells were also identified in the spinal cord of the animals belonging to the control group, as well as the presence of tissue around the cysts structuring the glial scar. Treatment with PPy/I or PPy/I+SW/EE reduced structural nerve tissue damage in the rostral and caudal portions of the injured spinal cord compared to the untreated control group. Moreover, in the group that received PPy/I treatment, some neurons under good conditions were observed in the rostral zone of the spinal cord, while in the group that received PPy/I+SW/EE treatment, the presence of neurons was observed in the rostral and caudal regions (Figure 9). The morphometric analysis showed that in the animals of the groups that received PPy/I or PPy/I+SW/EE (*p* < 0.05), the nervous tissue is better preserved after SCI (Figure 9).

### 3.4. Immunofluorescence analysis

**Figure 10:**
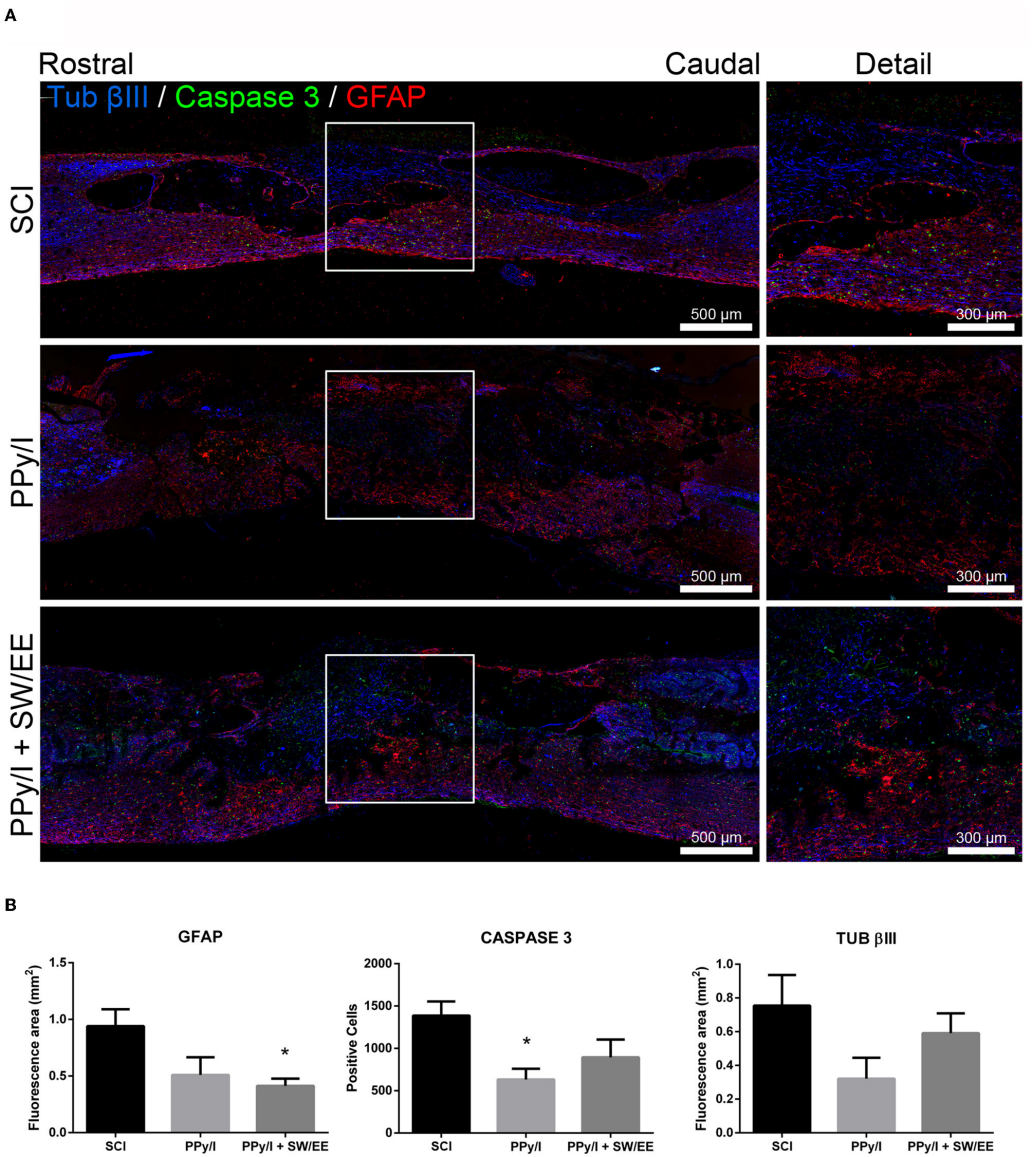
Longitudinal sections of the spinal cord of a representative animal from each of the 3 groups studied after spinal cord injury (SCI). Control without treatment (SCI), with polypyrrole-Iodine (PPy/I) synthesized by plasma and the combined treatment of PPy/I with a mixed scheme of rehabilitation by swimming and enriched environment (PPy/I+SW/EE). **(A)** Immunoflurescence analysis of GFAP (green), caspase-3 (red) and β-III tubulin (blue) expression. **(B)** GFAP expression increased in SCI group, while in the PPy/I+SW/EE group expression of GFAP decreased (*p* < 0.05). In PPy/I group the expression of caspase 3 was lower than in the control SCI group (*p* < 0.05). The expression of β-III tubulin did not have statistical differences between the 3 experimental groups. One-way ANOVA followed by a Tukey *post hoc* test *p < 0.05.

In the control group, the expression of GFAP increased in response to SCI due to reactive gliosis produced mainly by hypertrophic astrocytes (Figure 10A), while in the group treated with PPy/I+SW/EE, GFAP expression decreased (*p* < 0.05) at the interface between the biopolymer and spinal cord tissue (Figure 10B). The expression of caspase-3 (Figure 10A) in the spinal cord of the animals treated with PPy/I was lower than that observed in the control group (*p* < 0.05) (Figure 10B). The expression of β-III tubulin (Figure 10A) did not have statistical differences among the three experimental groups after 1 month of follow-up (Figure 10B).

## 4. Discussion

Although there is still no effective treatment for SCI in humans, the therapeutic combination of different strategies may promote better functional recovery. Nevertheless, the knowledge of the mechanisms of action of those strategies is necessary to find the best and most effective combinations.

Despite the fact that in the review by Venkatesh et al. (43), cell transplantation therapy is considered the most promising therapeutic strategy for the treatment of SCI; different biomaterials have also been reported as a good therapeutic option when used as scaffolds and embedded with growth factors and/or different types of cells (neural stem cell, embryonic/ pluripotent stem cells, mesenchymal/hematopoietic stem cells, oligodendrocytes, astrocytes, Schwann cells, and olfactory ensheathing cells). Alginate, chitosan channel-based, and collagen tube-based scaffolds are examples of this potential since these therapeutic approaches not only provide structural support but also offer a neurotrophic microenvironment (44). However, the use of biomaterials alone had not been considered.

Plasma-synthesized PPy /I can by itself neuroprotect tissue at the site of injury and prevent the progression of damage after experimental SCI (21), reduce inflammation (17), increase nerve conduction (20), limit glial scar formation by decreasing GFAP expression and, at the same time, significantly modifies the expression of molecules involved in the central nervous system regeneration and plasticities such as βIII-tubulin, myelin basic protein (MBP), and Gap-43 (19). This strategy also improves the general health of the animals and allows better recovery of motor function 2 months after SCI (17, 19).

Meanwhile, RB, as the most widely accepted treatment for patients with SCI, has been associated with some degree of functional recovery, preventing the loss of muscle tone, reducing spasticity, improving blood circulation, and increasing the expression of regeneration-related molecules (8–11). Although RB does not allow complete recovery of lost functions, its beneficial effects may be increased when combined with other therapeutic strategies such as the PPy/I application. In this sense, our research group has shown that the combination of PPy/I with RB through treadmill (17) or SW/EE (19) training allows for better recovery of motor function after a concussion, as seen in the present study. However, despite these findings, in-depth knowledge of the possible mechanisms for these strategies is still scarce.

Several studies using microarray analysis have described gene expression changes after SCI and showed that the microenvironment is seriously altered along with massively increased expression of proteins related to inflammation, oxidative stress, and cell death (45), while the genes of structural proteins of cells, constituents of extracellular matrix, and those encoding proteins involved in neurotransmission are downregulated after SCI (46).

In the present study, the analysis carried out using microarrays allowed us to identify the modifications in gene expression generated by PPy/I and PPy/I+SW/EE and to propose possible mechanisms of action of the treatments applied after SCI. Results showed that treatments modified the expression of several genes whose regulation seems to be associated with better recovery of motor function after SCI, many of which are involved in processes related to cell development. This finding suggests that promoting processes related to the development of the central nervous system is essential to design approaches for functional spinal cord repair.

Our research group previously showed that if after SCI, the microenvironment at the site of injury is appropriately modified by modulating the inflammatory response and limiting glial scar formation both, regeneration processes and functional recovery can be favored (19). Nevertheless, the mere modulation of the microenvironment may be insufficient to achieve the desired regeneration outcomes (47), and stimulating the intrinsic growth ability of mature neurons becomes relevant (48) since the decrease in the intrinsic regenerative capacity of mature neurons seems to be the main cause of regeneration failure after SCI (49).

In a model developed in Xenopus laevis, the expression of genes and biological processes that are activated in SCI was identified by comparing regenerative states against non-regenerative stages, and it was found that where regeneration occurs, genes related to neurogenesis and genes that promote axonal cone growth are expressed (50). Thus, neurogenesis and axonal regeneration have been proposed to contribute to the regenerative process after a massive loss of neurons and glia as well as the interruption of axonal tracts (50, 51). In this sense, treatments with PPy/I and PPy/I+SW/EE regulate processes related to neuronal development, neurogenesis, and neuronal differentiation, which is relevant since the spinal neurons that are lost during and after the injury need to be replaced in order to rebuild the local neuronal circuits.

Although stem cell transplantation has been reported to achieve certain success after SCI (52, 53), the identity of transplanted cells and restoration of functional circuitry in the injury site still require further investigation (54). Even more, the issue of neurogenesis in the spinal cord is still controversial, since in mammals, it has been shown that ependymal cells have neural stem cells or progenitor cell activity, but due to the microenvironment generated after SCI, these only give rise to astrocytes and oligodendrocytes and not to new neurons (55). In the current study, the findings of gene expression regarding possible neurogenesis showed that PPy/I modified 24 genes related to cellular processes, in general, some of them involved in developmental processes, biogenesis, synapses, and synaptic vesicle trafficking. This finding suggests that one of the possible mechanisms of action of PPy/I could include neuritogenesis and synaptic plasticity, which may in turn favor the reestablishment of synapses in surviving neurons. However, more studies are needed on the migration of immature neural cells from other neurogenic niches such as the meninges (56), or cells such as astrocytes in states of transdifferentiation (57) and whose pathways could converge at some point with neurogenesis at the site of injury, for example, it has been reported that NeuroD1, a neural transcription factor, can convert reactive astrocytes into neurons in the dorsal horn of stab-injured spinal cord with approximately 95% efficiency (58).

Our results also showed that individual treatment with PPy/I increased Tubb3 expression and motor function recovery 1 month after SCI. Tubb3 is considered a specific pan-neuronal marker (59), the expression of which is high during the developmental period in the central nervous system where axonal guidance and maturation occur. It has been described that the expression of Tubb3 is greater during the period of development where axonal guidance and ripening take place and that the levels of Tubb3 decrease in the adult central nervous system, although remain elevated in the peripheral nervous system (4). Nevertheless, the Tubb3 expression in adulthood remains constant in neurons as a structural component of the microtubules of the cytoskeleton (4, 60). Different studies indicate that mutations in Tubb3 produce alterations in important axonal tracts of the nervous system. Therefore, Tubb3 has a specific function for the development of the nervous system and the maintenance of the axons (7, 61–63), and in the case of central nervous system injuries, Tubb3 could have a preponderant function in the maintenance of the cytoarchitecture and/or in a possible functional recovery. Thus, the increase in the Tubb3 expression is considered a marker of neuroregeneration.

In the present study, although the treatment with PPy/I+SW/EE increased the expression of the Tubb4 gene and treatment with PPy/I significantly increased the expression of the Tubb3 gene, identification of the βIII-tubulin protein by immunohistochemical techniques in the spinal cord tissue did not show significant differences between the control group and the groups that received treatments 1 month after SCI. This may be due to the inhibition of Tubb3 transduction at this specific time post-SCI. Inhibitors of protein expression have been identified in neuronal tissues, including ID2 and Pax3, which seem to be able to repress Tubb3 transcription (64, 65). In rat neuronal stem cells, Pax3 can bind to the Tubb3 promoter regions and inhibit transcription and translation (65). However, the inhibition of Tubb3 in our model can disappear later, since in a study that our research group carried out 2 months after SCI, a significant increase in the βIII-tubulin expression was observed in the PPy/I and PPy/I+SW/EE-treated groups [19]. Furthermore, in that study, our group showed that βIII-tubulin co-localized with Gap-43, a protein related to nerve regeneration, which could mean that it is important not only to induce neurons in the growth mode but to maintain that state of growth to have a clinically relevant effect.

Tubb4, as a member of the tubulin family, is specifically expressed in oligodendrocytes and assembled into the microtubule network during the premyelination stage and initial myelination. Moreover, the predominant isoform during the myelination stage in postnatal oligodendrocytes is the Tubb4a isotype (66). Thus, the increase in Tubb4 can reduce myelination defects and decrease dysfunction in neuronal connectivity (67). As the combined treatment with PPy/I+SW/EE also increased the expression of Tubb4a, this could also explain the improvement in motor activity at end of follow up. In a previous study, an increase in MBP was observed at the injury site 2 months after treatment (19), which suggests that combined treatment with PPy/I+SW/EE favors myelination processes.

It has been suggested that after SCI, therapeutic strategies that allow changing the cellular environment of the CNS to one that is permissive and promotes nerve regeneration will be those that result in the recovery of lost functions. Following SCI, neuronal plasticity may be blocked as astrocytes and microglia form the glial scar, resulting in an increased expression of molecules such as GFAP. The problem is that the glial scar can limit axonal growth by constituting a physical barrier that prevents neural and axonal growth (68) and activated astrocytes upregulate complement cascade genes, destroy synapses, decrease their phagocytic capacity, and induce apoptosis in neurons and oligodendrocytes (69, 70) that affect recovery following SCI. In the present study, treatment with PPy/I+SW/EE decreased GFAP expression and glial scar formation, which perhaps allowed the fibers identified at the epicenter of the lesion to connect with those that survived both in the rostral region and caudal region of the spinal cord and this favored the recovery of motor function in the animals of this group, contrary to what was observed in the control group.

Caspase-3 is a proenzyme that acts as an inducible cell death effector in both neurons and oligodendrocytes after SCI (71). In the present study, PPy/I treatment reduces caspase-3 expression, which perhaps helped to positively modify the microenvironment at the lesion site because it has been described that high levels of caspase-3 reduce the expression of NGF after SCI and that the decrease of this proenzyme increases its expression (72).

In the present study, the PPy/I+SW/EE group received the best functional recovery, since this therapeutic combination could enhance their beneficial effects through a pro-angiogenic effect generated by the increase in the gene expression of VEGFb, a vital factor in angiogenesis, neuronal development, and neuronal regeneration (73). Fourteen days after SCI, tissue levels of VEGF mRNA and its protein increase and eventually normalize, but reduced VEGF expression after SCI impairs neurogenesis and angiogenesis, which are required to promote neuroprotection and survival of injured nerve tissue (74). In most tissues, particularly neural tissues, including the retina, brain, and spinal cord, VEGF isotype β is observed at high levels (75). Moreover, the participation of VEGFβ in the regulation of neurogenesis in the adult brain was demonstrated in knockout mice for VEGFβ, which presents reduced neurogenesis (76, 77). Thus, VEGFβ represents a potent survival and protective factor for motor, cortical, retinal, and spinal cord neurons (77, 78). In addition to the above, Zeng et al. showed that recombinant VEGF-neural stem cell transplantation after SCI is more effective than normal neural stem cell transplantation alone (79). Furthermore, it has been shown that treatments that stimulate angiogenic processes, oxygenation, and blood circulation promote the preservation and recovery of nerve tissue and motor function (80, 81). Because blood vessel permeability may increase after SCI, this may have harmful effects on functional recovery (82), whereas the promotion of vascular stabilization and reduction of lesion volume may promote better functional recovery in the chronic phase of SCI (83). Here, it was demonstrated that PPy/I+SW/EE favor the upregulation of VEGFβ, which stimulates embryonic angiogenesis, and probably the combination of both strategies allows an adequate conformation of the blood vessel networks, which results in a better functional recovery when compared to the application of a single strategy.

It had also been reported about significant alterations in SLC17A7 (VGLUT1) expression from 1 h to 3 months after SCI (84). SLC17A7 belongs to a big family of sialic acid proteins that are known to play an important role in absorbing glutamate into synaptic vesicles at presynaptic nerve terminals and excitatory neurons (85–87). Thus, their promotion could be relevant in neuronal damage control and functional recovery. Our findings show that the administration of PPy/I alone increases the expression of Scl29a2, Scl32a1, and Scl8a3, as well as other isoforms of sialic acid proteins, 1 month after SCI, while PPy/I+SW/EE treatment increases the expression of Scl8a3, Slc8a5, Slc9a1, Scl16a3, Scl35a12, and Scl35b3. The GO analysis highlights that protein members of the sialic acid family also participate in remodeling cellular components, such as neuronal cell body, neuron projection terminals, and presynapse, which partially can explain why animals that receive this treatment have better and faster functional recovery. In addition to the above, combined treatment with PPy/I+SW/EE has upregulated genes involved in remodeling the plasma membrane and potassium channel voltage located in the somatodendritic compartments such as Kcng4 and Kcnc3.

Other studies have shown a continued decrease in the gene expression of members of the ADAM family that affects neuronal plasticity at the chronic stage of SCI (84). In animals from the PPy/I group, an upregulated expression of ADAM8, a member of ADAM's family, was observed. ADAM8 can interact with other molecules, such as calcium channels (Cacna1b), which was highly expressed and can support synapse formation and maturation between presynaptic and postsynaptic structures. Furthermore, ADAM8 plays a role in the proliferation and/or migration of endothelial cells during angiogenesis following SCI (88) through the modulation of a complex interplay between different proteins including VEGF.

Similar to our study, other authors found that the expression of genes related to neuroplasticity and angiogenesis was upregulated by RB (2). In the DAVID database, the most functional annotation clustering to the PPy/I+SW/EE treatment included genes involved in angiogenesis, oxygenation, and blood circulation, all of which are required to preserve and recover nerve tissue and, with it, motor function.

Because the underlying molecular mechanisms are still not fully understood, research in this area must continue in order to understand them and to positively influence them.

## 5. Conclusion

Our results provide a view of how PPy/I and PPy/I+SW/EE treatments could allow spinal cord regeneration, and which genes in particular are being regulated. This is valuable because the knowledge of how different cellular and molecular processes contribute to spinal cord regeneration allows us to consider their modulation through new strategies to promote the regeneration of the mammalian spinal cord and its functionality.

PPy/I modifies the expression of genes involved in the developmental process, biogenesis, synapse, and synaptic vesicle trafficking that could induce neurogenesis and synaptic plasticity.

PPy/I+SW/EE promotes the expression of genes involved in the developmental processes, proliferation, neuron development, morphogenesis, cell differentiation, neurogenesis biogenesis, synapses, and synaptic vesicle trafficking.

Although the expression of β-III tubulin was observed in all groups, a decreased expression of caspase-3 in the PPy/I group and GFAP in the PPy/I+SW/EE group was demonstrated, which improve neuronal survival by producing modifications that generate a pro-regenerative microenvironment and improving motor functional recovery in rats with SCI.

Better preservation of nerve tissue was observed in PPy/I and PPy/SW/EE groups.

The best motor function recovery after SCI was observed in the PPy/I+SW/EE group 1 month after follow-up. Thus, this treatment could represent a therapeutic alternative for SCI.

## Data availability statement

The datasets presented in this study can be found in online repositories. The names of the repository/repositories and accession number(s) can be found in the article/Supplementary material.

## Ethics statement

The animal study was reviewed and approved by National Scientific Research Commission of the Mexican Social Security Institute. Registration number 2015-785-060.

## Author contributions

HS-C, SS-T, AC-S, and CO-B: conceptualization, writing-original draft preparation, and writing-review and editing. SS-T, HS-C, AC-S, CO-B, LA-M, RM-L, MO, GC, RO, JM-C, AM-G, AD-R, CR, OF-S, and AA-G: investigation. HS-C: supervision. All authors have read and agreed to the published version of the manuscript.
