# Supplementary material for: Gene expression and locomotor recovery in adult rats with spinal cord injury and plasma-synthesized polypyrrole/iodine application combined with a mixed rehabilitation scheme
Source: Front Neurol. 2023 May 23;14:1124245. doi: 10.3389/fneur.2023.1124245 (PMC10243140; doi:10.3389/fneur.2023.1124245)
Supplement: Supplementary file 1 [file Data_Sheet_1.docx]

Supplementary Material

**GENE EXPRESSION AND LOCOMOTOR RECOVERY IN ADULT RATS WITH SPINAL CORD INJURY AND PLASMA-SYNTHESIZED POLYPYRROLE/IODINE APPLICATION COMBINED WITH A MIXED REHABILITATION SCHEME**

Angélica Coyoy-Salgado^1,3‡^, Carlos Orozco-Barrios^1,3‡^, Stephanie Sánchez-Torres^2,3^, María Guadalupe Olayo^4^, Guillermo Jesus Cruz^4^, Juan Morales-Corona^5^, Roberto Olayo^5^, Araceli Diaz-Ruiz^6^, Camilo Ríos^6^, Laura Alvarez-Mejia^2,3^, Rodrigo Mondragón-Lozano^1,3^, Axayacatl Morales-Guadarrama^5^, Ana Lucía Alonso García^7^ Omar Fabela-Sánchez^8^, and Hermelinda Salgado-Ceballos^2,3,*^

*** Correspondence:** Dr. Hermelinda Salgado-Ceballos (H S-C), e-mail: melisalce@yahoo.com

**Table S1. Genes expressed upstream (65) and expressed downstream (17) after spinal cord injury and PPy/I administration**

| **UP** | | | |
| --- | --- | --- | --- |
| **Gene Symbol** | **Description** | **Fold Change** | **P-Val** |
| Ncbp2; Pigz; | Nuclear cap binding protein subunit 2 | 7.77 | 0.0041 |
| Pigz | Phosphatidylinositol glycan anchor biosynthesis, class Z | 6.34 | 0.0049 |
| LOC102552104 | MLV-related proviral Env polyprotein-like | 4.66 | 0.0187 |
| LOC102555039 | MLV-related proviral Env polyprotein-like | 4.66 | 0.0187 |
| Kcnc3 | Potassium channel, voltage gated Shaw-related subfamily C, member 3 | 4.08 | 0.0106 |
| Masp1 | Mannan-binding lectin serine peptidase 1 | 3.46 | 0.0009 |
| LOC102555324 | MLV-related proviral Env polyprotein-like | 3.19 | 0.0431 |
| Plppr2 | Phospholipid phosphatase related 2 | 3.15 | 0.0204 |
| Lynx1 | Ly6/neurotoxin 1 | 2.97 | 0.0122 |
| Slc32a1 | Solute carrier family 32 (GABA vesicular transporter), member 1 | 2.84 | 0.0333 |
| Mab21l2 | Mab-21-like 2 (C. elegans) | 2.77 | 0.0101 |
| Sgpp2 | Sphingosine-1-phosphate phosphatase 2 | 2.73 | 0.0133 |
| Vgf | VGF nerve growth factor inducible | 2.72 | 0.0018 |
| LOC102553221 | MLV-related proviral Env polyprotein-like | 2.64 | 0.0331 |
| Ubald1 | UBA-like domain containing 1 | 2.61 | 0.0086 |
| Ache | Acetylcholinesterase; UFM1-specific peptidase 1 | 2.57 | 0.0096 |
| Syt3 | Synaptotagmin III | 2.56 | 0.0118 |
| Pomgnt2 | Protein O-linked mannose N-acetylglucosaminyltransferase 2 (beta 1,4-) | 2.55 | 0.0019 |
| Fam189b | Family with sequence similarity 189, member B | 2.55 | 0.0107 |
| Nptxr | Neuronal pentraxin receptor | 2.53 | 0.0079 |
| Per1 | Period circadian clock 1 | 2.53 | 0.0119 |
| Dmtn | Dematin actin binding protein | 2.51 | 0.0105 |
| Bsn | Bassoon (presynaptic cytomatrix protein) | 2.51 | 0.0182 |
| Thy1 | Thy-1 cell surface antigen | 2.5 | 0.0189 |
| Nat8l | N-acetyltransferase 8-like | 2.47 | 0.0226 |
| Rph3a | Rabphilin 3A | 2.46 | 0.0227 |
| Rerg | RAS-like, estrogen-regulated, growth-inhibitor | 2.41 | 0.0024 |
| RGD1307443 | Similar to mKIAA0319 protein | 2.41 | 0.0300 |
| Sez6l2 | Seizure related 6 homolog (mouse)-like 2 | 2.39 | 0.0342 |
| Slc18a3 | Solute carrier family 18 (vesicular acetylcholine transporter), member 3 | 2.37 | 0.0033 |
| Brinp1 | Bone morphogenetic protein/retinoic acid inducible neural-specific 1 | 2.36 | 0.0140 |
| Prrt2 | Proline-rich transmembrane protein 2 | 2.33 | 0.0120 |
| Spock2 | Sparc/osteonectin, cwcv and kazal-like domains proteoglycan (testican) 2 | 2.32 | 0.0404 |
| Btbd11 | BTB (POZ) domain containing 11 | 2.32 | 0.0492 |
| Nptx1 | Neuronal pentraxin I | 2.28 | 0.0076 |
| Ncdn | neurochondrin | 2.24 | 0.0182 |
| Nefh | Neurofilament, heavy polypeptide | 2.24 | 0.0499 |
| Sst | Somatostatin | 2.21 | 0.0148 |
| Camkk1 | Calcium/calmodulin-dependent protein kinase kinase 1, alpha | 2.2 | 0.0096 |
| Tmem145 | Transmembrane protein | 2.2 | 0.0140 |
| Dnajc6 | DnaJ (Hsp40) homolog, subfamily C, member 6 | 2.17 | 0.0299 |
| Fam69b | Family with sequence similarity 69, member B | 2.16 | 0.0062 |
| Mest | Mesoderm specific transcript | 2.16 | 0.0169 |
| Shisa9 | Cystine-Knot AMPAR Modulating Protein | 2.15 | 0.0017 |
| Hapln4 | Hyaluronan and proteoglycan link protein | 2.15 | 0.0051 |
| RGD1305455 | Similar to hypothetical protein FLJ10925 | 2.15 | 0.0128 |
| Crtc1 | CREB regulated transcription coactivator 1 | 2.13 | 0.0382 |
| Slc29a2 | Solute carrier family 29 | 2.11 | 0.0004 |
| Pcsk2 | Proprotein convertase subtilisin/kexin type 2 | 2.11 | 0.0259 |
| Chgb | Chromogranin B | 2.11 | 0.0326 |
| Atp13a2 | ATPase type 13A2 | 2.11 | 0.0380 |
| Lgi2 | Leucine-rich repeat LGI family, member 2 | 2.09 | 0.0455 |
| Adam8 | ADAM metallopeptidase domain 8 | 2.08 | 0.0121 |
| Syngr3 | Synaptogyrin 3 | 2.08 | 0.0220 |
| Samd14 | Sterile alpha motif domain containing 14 | 2.08 | 0.0247 |
| Trpt1 | tRNA phosphotransferase 1 | 2.07 | 0.0295 |
| Gpr162 | G protein-coupled receptor 162 | 2.06 | 0.0273 |
| Phyhip | Phytanoyl-CoA 2-hydroxylase interacting protein (Phyhip) | 2.06 | 0.0440 |
| Wbp2 | WW domain binding protein 2 | 2.05 | 0.0397 |
| Tmem151a | Transmembrane protein 151A | 2.04 | 0.0350 |
| Tmem151a | Transmembrane protein 151A (Tmem151a) | 2.04 | 0.0350 |
| Clip3 | CAP-GLY domain containing linker protein 3 | 2.03 | 0.0170 |
| **Tubb3** | **Tubulin, beta 3 class III** | **2.03** | **0.0404** |
| Cygb | Cytoglobin | 2.02 | 0.0469 |
| Sncb | Synuclein, beta | 2.01 | 0.0419 |

| **DOWN** | | | |
| --- | --- | --- | --- |
| **Gene Symbol** | **Description** | **Fold Change** | **P-val** |
| Wapal | Protein Wapal; wings apart-like homolog (Drosophila) | -2.02 | 0.0006 |
| Lpar4 | Lysophosphatidic acid receptor 4 | -2.02 | 0.0352 |
| Calb1 | Calbindin 1 | -2.03 | 0.0083 |
| Stag1 | Stromal antigen 1 | -2.04 | 0.0430 |
| Cdk1 | Cyclin-dependent kinase 1 | -2.07 | 0.0116 |
| Tc2n | Tandem C2 domains, nuclear | -2.07 | 0.0313 |
| Dnhd1-ps1; LOC690093; Dnhd1 | Protein Dnhd1-ps1; dynein heavy chain domain 1, pseudogene 1; hypothetical protein LOC690093. | -2.08 | 0.0484 |
| Junb | Jun B proto-oncogene | -2.12 | 0.0043 |
| Dpysl3 | Dihydropyrimidinase-related protein 3 | -2.13 | 0.0260 |
| Cyr61 | Cysteine-rich, angiogenic inducer, 61 | -2.15 | 0.0255 |
| Dnah11 | Dynein, axonemal, heavy chain 11 | -2.17 | 0.0002 |
| Kitlg | KIT ligand | -2.17 | 0.0105 |
| Ccnl2 | Cyclin L2 | -2.24 | 0.0333 |
| LOC102551451 | Zinc finger protein 665-like | -2.25 | 0.0078 |
| Fhit | Fragile histidine triad gene (Fhit), mRNA. | -2.28 | 0.0206 |
| Pnisr | PNN-interacting serine/arginine-rich protein | -2.32 | 0.0239 |
| Arhgap15 | Rho GTPase activating protein 15 | -2.34 | 0.0034 |
| LOC500300 | Similar to hypothetical protein MGC6835 | -2.34 | 0.0142 |
| Wsb1 | WD repeat and SOCS box-containing 1 | -2.41 | 0.0265 |
| Erap1 | Endoplasmic reticulum aminopeptidase 1 | -2.5 | 0.0136 |
| LOC361346 | Similar to chromosome 18 open reading frame 54 | -2.53 | 0.0003 |
| Kank3 | KN motif and ankyrin repeat domains 3 | -2.53 | 0.0439 |
| Slc35b3 | Solute carrier family 35 (adenosine 3-phospho 5-phosphosulfate transporter), member B3 | -2.54 | 0.0015 |
| Akr1cl | Aldo-keto reductase family 1, member C-like | -2.58 | 0.0181 |
| Rsad2 | Radical S-adenosyl methionine domain containing 2 | -2.68 | 0.0024 |
| Slco1c1 | Solute carrier organic anion transporter family, member 1c1 | -2.69 | 0.0382 |
| Dnah12 | Dynein, axonemal, heavy chain 12. | -2.77 | 0.0072 |
| Adgrl4 | Adhesion G protein-coupled receptor L4 | -2.78 | 0.0054 |
| NONMMUG024767 | Non-coding transcript identified by NONCODE: Exonic | -2.98 | 0.0178 |
| Dopey1 | Dopey family member 1 | -3.08 | 0.0112 |
| Rgs5 | Regulator of G-protein signaling 5 | -3.21 | 0.0041 |
| Hbb-b1; LOC689064 | Hemoglobin, beta adult major chain. | -3.44 | 0.0019 |
| Gpcpd1 | Glycerophosphocholine phosphodiesterase GDE1 homolog (S. cerevisiae) | -3.72 | 0.0115 |
| LOC689064 | Beta-globin | -3.82 | 0.0091 |
| LOC689064 | Beta-globin (LOC689064), mRNA | -4.03 | 0.0028 |
| Hba2; Hba1 | Hemoglobin, alpha 2; hemoglobin, alpha 1 | -4.05 | 0.0014 |
| Hba1 | Hemoglobin, alpha 1 | -4.22 | 0.0056 |
| Slc39a12 | Solute carrier family 39 (zinc transporter), member 12 | -4.31 | 0.0471 |
| Hbb-b1 | Hemoglobin, beta adult major chain | -4.49 | 0.0018 |
| NONMMUG002175 | Non-coding transcript identified by NONCODE: Exonic | -4.83 | 0.0144 |
| LOC100134871 | Beta globin minor gene | -4.85 | 0.0012 |
| Hbb | Hemoglobin, beta | -5.59 | 0.0007 |
| Hba-a1 | Hemoglobin alpha, adult chain 1 | -5.65 | 0.0005 |

**Table 2. Genes expressed upstream (41) and expressed downstream (43) after spinal cord injury and PPy/I + SW/EE treatment**

| **UP** | | | | |
| --- | --- | --- | --- | --- |
| **Gene Symbol** | **Description** | **Fold Change** | **P-val** |  |
| Kcnc3 | Potassium channel, voltage gated Shaw-related subfamily C, member 3 | 3.48 | 0.0469 |  |
| Kcng4 | Potassium channel, voltage gated modifier subfamily G, member 4 | 3.44 | 0.0183 |  |
| Unc5b | Unc-5 netrin receptor B | 3.26 | 0.0195 |  |
| Clip3 | CAP-GLY domain containing linker protein 3 | 2.6 | 0.0141 |  |
| Ttyh3 | Tweety family member 3 | 2.51 | 0.0278 |  |
| LOC102552104 | MLV-related proviral Env polyprotein-like | 2.48 | 0.0349 |  |
| LOC102555039 | MLV-related proviral Env polyprotein-like | 2.48 | 0.0349 |  |
| Sez6 | Seizure related 6 homolog (mouse) | 2.47 | 0.0293 |  |
| Plekho2 | Pleckstrin homology domain containing, family O member 2 | 2.46 | 0.0226 |  |
| Wasf1 | WAS protein family, member 1 | 2.44 | 0.0294 |  |
| Dpysl5 | Protein LOC100911610; ENCODES a protein that exhibits hydrolase activity acting on carbon-nitrogen (but not peptide) bonds in cyclic amides | 2.43 | 0.0055 |  |
| Plppr2 | Phospholipid phosphatase related 2 | 2.33 | 0.0422 |  |
| Slc16a3 | Solute carrier family 16 (monocarboxylate transporter), member 3 | 2.31 | 0.0067 |  |
| Cacna1b | Calcium channel, voltage-dependent, N type, alpha 1B subunit | 2.31 | 0.0160 |  |
| Fam189b | Family with sequence similarity 189, member B | 2.31 | 0.0424 |  |
| Cfap43; LOC690101; Wdr96 | Cilia and flagella associated protein 43; | 2.28 | 0.0205 |  |
| Lpcat4 | Lysophosphatidylcholine acyltransferase 4 | 2.27 | 0.0292 |  |
| Myrf | Myelin regulatory factor | 2.26 | 0.0395 |  |
| Brinp1 | Bone morphogenetic protein/retinoic acid inducible neural-specific 1 | 2.21 | 0.0386 |  |
| Slc18a3 | Solute carrier family 18 (vesicular acetylcholine transporter), member 3 | 2.2 | 0.0356 |  |
| Tor1aip1 | Torsin A interacting protein 1 | 2.18 | 0.0362 |  |
| **Tubb4a** | **Tubulin, beta 4A class IVa** | **2.18** | **0.0385** |  |
| Gp1bb; Sept5 | Glycoprotein Ib (platelet), beta polypeptide; septin 5 | 2.17 | 0.0245 |  |
| Paqr4 | Progestin and adipoQ receptor family member IV | 2.16 | 0.0141 |  |
| Slc17a5 | Solute carrier family 17 (acidic sugar transporter), member 5 | 2.15 | 0.0344 |  |
| Slc36a1 | Solute carrier family 36 (proton/amino acid symporter), member 1 | 2.14 | 0.0027 |  |
| Olr768 | Olfactory receptor 768 | 2.14 | 0.0104 |  |
| Parvg | Parvin, gamma | 2.13 | 0.0413 |  |
| Bcl2 | B-cell CLL/lymphoma 2 | 2.12 | 0.0474 |  |
| Cpne5 | Copine V | 2.09 | 0.0198 |  |
| Slc9a1 | Solute carrier family 9, subfamily A (NHE1, cation proton antiporter 1), member 1 | 2.09 | 0.0236 |  |
| Hapln4; LOC100911378 | Hyaluronan and proteoglycan link protein 4 | 2.07 | 0.0126 |  |
| Aoah | Acyloxyacyl hydrolase (neutrophil) | 2.05 | 0.0021 |  |
| **Vegfb** | **Vascular endothelial growth factor B** | **2.04** | **0.0329** |  |
| Cntfr | Ciliary neurotrophic factor receptor | 2.04 | 0.0414 |  |
| Mgat3 | Mannosyl (beta-1,4-)-glycoprotein beta-1,4-N-acetylglucosaminyltransferase | 2.03 | 0.0022 |  |
| Pou6f1 | POU class 6 homeobox 1 | 2.03 | 0.0166 |  |
| Cdkn1a | Cyclin-dependent kinase inhibitor 1A | 2.01 | 0.0026 |  |
| Pomgnt2 | Protein O-linked mannose N-acetylglucosaminyltransferase 2 (beta 1,4-) | 2 | 0.0217 |  |
| Spen | Spen family transcriptional repressor | 2 | 0.0226 |  |
| Gpx3 | Glutathione peroxidase 3 | 2 | 0.0437 |  |

| **DOWN** | | | |
| --- | --- | --- | --- |
| **Gene Symbol** | **Description** | **Fold Change** | **P-val** |
| Wapal | Protein Wapal; wings apart-like homolog (Drosophila) | -2.02 | 0.0006 |
| Lpar4 | Lysophosphatidic acid receptor 4 | -2.02 | 0.0352 |
| Calb1 | Calbindin 1 | -2.03 | 0.0083 |
| Stag1 | Stromal antigen 1 | -2.04 | 0.0430 |
| Cdk1 | Cyclin-dependent kinase 1 | -2.07 | 0.0116 |
| Tc2n | Tandem C2 domains, nuclear | -2.07 | 0.0313 |
| Dnhd1-ps1; LOC690093; Dnhd1 | Protein Dnhd1-ps1; dynein heavy chain domain 1, pseudogene 1; hypothetical protein LOC690093. | -2.08 | 0.0484 |
| Junb | Jun B proto-oncogene | -2.12 | 0.0043 |
| Dpysl3 | Dihydropyrimidinase-related protein 3 | -2.13 | 0.0260 |
| Cyr61 | Cysteine-rich, angiogenic inducer, 61 | -2.15 | 0.0255 |
| Dnah11 | Dynein, axonemal, heavy chain 11 | -2.17 | 0.0002 |
| Kitlg | KIT ligand | -2.17 | 0.0105 |
| Ccnl2 | Cyclin L2 | -2.24 | 0.0333 |
| LOC102551451 | Zinc finger protein 665-like | -2.25 | 0.0078 |
| Fhit | Fragile histidine triad gene (Fhit), mRNA. | -2.28 | 0.0206 |
| Pnisr | PNN-interacting serine/arginine-rich protein | -2.32 | 0.0239 |
| Arhgap15 | Rho GTPase activating protein 15 | -2.34 | 0.0034 |
| LOC500300 | Similar to hypothetical protein MGC6835 | -2.34 | 0.0142 |
| Wsb1 | WD repeat and SOCS box-containing 1 | -2.41 | 0.0265 |
| Erap1 | Endoplasmic reticulum aminopeptidase 1 | -2.5 | 0.0136 |
| LOC361346 | Similar to chromosome 18 open reading frame 54 | -2.53 | 0.0003 |
| Kank3 | KN motif and ankyrin repeat domains 3 | -2.53 | 0.0439 |
| Slc35b3 | Solute carrier family 35 (adenosine 3-phospho 5-phosphosulfate transporter), member B3 | -2.54 | 0.0015 |
| Akr1cl | Aldo-keto reductase family 1, member C-like | -2.58 | 0.0181 |
| Rsad2 | Radical S-adenosyl methionine domain containing 2 | -2.68 | 0.0024 |
| Slco1c1 | Solute carrier organic anion transporter family, member 1c1 | -2.69 | 0.0382 |
| Dnah12 | Dynein, axonemal, heavy chain 12. | -2.77 | 0.0072 |
| Adgrl4 | Adhesion G protein-coupled receptor L4 | -2.78 | 0.0054 |
| NONMMUG024767 | Non-coding transcript identified by NONCODE: Exonic | -2.98 | 0.0178 |
| Dopey1 | Dopey family member 1 | -3.08 | 0.0112 |
| Rgs5 | Regulator of G-protein signaling 5 | -3.21 | 0.0041 |
| Hbb-b1; LOC689064 | Hemoglobin, beta adult major chain. | -3.44 | 0.0019 |
| Gpcpd1 | Glycerophosphocholine phosphodiesterase GDE1 homolog (S. cerevisiae) | -3.72 | 0.0115 |
| LOC689064 | Beta-globin | -3.82 | 0.0091 |
| LOC689064 | Beta-globin (LOC689064), mRNA | -4.03 | 0.0028 |
| Hba2; Hba1 | Hemoglobin, alpha 2; hemoglobin, alpha 1 | -4.05 | 0.0014 |
| Hba1 | Hemoglobin, alpha 1 | -4.22 | 0.0056 |
| Slc39a12 | Solute carrier family 39 (zinc transporter), member 12 | -4.31 | 0.0471 |
| Hbb-b1 | Hemoglobin, beta adult major chain | -4.49 | 0.0018 |
| NONMMUG002175 | Non-coding transcript identified by NONCODE: Exonic | -4.83 | 0.0144 |
| LOC100134871 | Beta globin minor gene | -4.85 | 0.0012 |
| Hbb | Hemoglobin, beta | -5.59 | 0.0007 |
| Hba-a1 | Hemoglobin alpha, adult chain 1 | -5.65 | 0.0005 |

**Table S3.** Differential gene expression profile after Spinal Cord Injury (SCI) treated with plasma synthesized polypyrrole/iodine (PPy/I) vs SCI.

1. **Molecular Function**

|  | **#** | **%H** | **Gene** |
| --- | --- | --- | --- |
| Binding (GO:0005488) | 15 | 44.1 | Lynx1, Dnajc6, Ncbp2, Dapl1, Rbbp8, Tubb3, Crtc1, Rerg  Mnd1, Ccl9, Dmtn, Per1, Samd14, Wbp2, Syt3 |
| Catalytic activity (GO:0003824) | 9 | 26.5 | Plppr2, Masp1, Mettl16, Pigz, Pcsk2, Rbbp8, Tpk1, Rerg  Camkk1 |
| Molecular function regulator (GO:0098772 | 2 | 5.9 | Lynx1, Ccl9 |
| Molecular transducer activity (GO:0060089) | 1 | 2.9 | Lynx1 |
| Structural molecule activity (GO:0005198) | 1 | 2.9 | Tubb3 |
| Transcription regulator activity (GO:0140110) | 1 | 2.9 | Wbp2 |
| Transporter activity (GO:0005215) | 5 | 14.7 | Slc29a2, Rerg, Kcnh2, Slc32a1, Kcnc3, Slc18a3 |

1. **Biological process**

|  | **#** | **%H** | **Gene** |
| --- | --- | --- | --- |
| Biological phase (GO:0044848) | 1 | 1 | Mnd1 |
| Biological regulation (GO:0065007) | 15 | 14.7 | Sst, Pcsk2, Ache, Dapl1, Atp13a2, Kcnh2, Crtc1, Rerg, Ccl9, Camkk1, Per1, Samd14, Wbp2, Brinp1, Syt3 |
| Cellular component organization or biogenesis (GO:0071840) | 8 | 7.8 | Mettl16, Dnajc6, Tubb3, Mnd1, Dmtn, Samd14, Brinp1, Syt3 |
| Cellular process (GO:0009987) | 24 | 23.5 | Mettl16, Dnajc6, Pigz, Sst, Ncbp2., Pcsk2, Ache, Dapl1, Atp13a2, Rbbp8, Tpk1, Crtc1, Tubb3, Rerg, Mnd1, Cccl9, Dmtn, Camkk1, Per1, Samd14, Wbp2, Slc8a3, Brinp1, Syt3 |
| Developmental process (GO:0032502) | 3 | 2.9 | Hapln4, Samd14, Brinp1 |
| Immune system process (GO:0002376) | 1 | 1 | Ccl9 |
| Localization (GO:0051179) | 9 | 8.8 | Slc29a2, Dnajc6, Sst, Ache, Rerg, Slc32a1, Ccl9, Kcnc3, Syt3 |
| Locomotion (GO:0040011) | 2 | 2 | Sst, Cccl9 |
| Metabolic process (GO:0008152) | 12 | 11.8 | Mettl16, Pigz, Ncbp2, Pcsk2, Dapl1, Rbbp8, Tpk1, Crtc1, Mnd1, Cccl9, Per1, Wbp2 |
| Multi-organism process (GO:0051704) | 1 | 1 | Ccl9 |
| Multicellular organismal process (GO:0032501) | 5 | 4.9 | Ache, Hapln4, Per1, Samd14, Brinp1 |
| Reproduction (GO:0000003) | 1 | 1 | Mnd1 |
| Reproductive process (GO:0022414) | 1 | 1 | Mnd1 |
| Response to stimulus (GO:0050896) | 10 | 9.8 | Ache, Dapl1, Rbbp8, Rerg, Cccl9, Camkk1, Per1, Samd14, Brinp1, Syt3 |
| Rhythmic process (GO:0048511) | 1 | 1 | Per1 |
| Signaling (GO:0023052) | 8 | 7.8 | Ache, Dapl1, Rerg, Cccl9, Camkk1, Samd14, Slc8a3, Syt3 |

1. **Cellular component**

|  | **#** | **%H** | **Gene** |
| --- | --- | --- | --- |
| Cell part (GO:0044464) | 25 | 20.5 | Chgb, Slc29a2, Lynx1, Dnajc6, Pigz, Syngr3, Ncbp2, Pcsk2, Chrne, Rbbp8, Kcnh2, Tubb3, Crtc1, Rerg, Slc32a1, Mnd1, Dmtn, Camkk1, Kcnc3, Per1, Samd14, Wbp2, Slc18a3, Brinp1, Syt3 |
| Cell (GO:0005623) | 25 | 20.5 | Chgb, Slc29a2, Lynx1, Dnajc6, Pigz, Syngr3, Ncbp2, Pcsk2, Ache, Rbbp8, Tubb3, Crtc1, Rerg, Slc32a1, Mnd1, Dmtn, Camkk1, Kcnc3, Per1, Samd14, Wbp2, Slc18a3, Brinp1, Syt3 |
| Extracellular region part (GO:0044421) | 6 | 4.9 | Chgb, Masp1, Sst¸Pcsk2, Ccl9, Hapln4 |
| Extracellular region (GO:0005576) | 6 | 4.9 | Chgb, Masp1, Sst¸Pcsk2, Ccl9, Hapln4 |
| Membrane part (GO:0044425) | 10 | 8.2 | Slc29a2, Lynx1, Pcsk2, Ache, Rerg, Slc32a1, Kcnc3, Samd14, Wbp2, Slc18a3, Brinp1 |
| Membrane (GO:0016020) | 15 | 12.3 | Chgb, Slc29a2, Lynx1, Pigz, Syngr3, Pcsk2, Ache, Rerg, Slc32a1, Kcnc3, Samd14, Slc18a3, Brinp1, Syt3 |
| Organelle part (GO:0044422) | 6 | 4.9 | Syngr3, Pcsk2, Rbbp8, Tubb3, Slc32a1, Slc18a3, |
| Organelle (GO:0043226) | 17 | 13.9 | Chgb, Dnajc6, Pigz, Syngr3, Pcsk2, Rbbp8, Tubb3, Crtc1, Slc32a1, Mnd1, Dmtn, Per1, Samd14, Wbp2, Slc18a3, Brinp1, Syt3 |
| Protein-containing complex (GO:0032991) | 3 | 2.5 | Ncbp2, Kcnc3, Slc18a3 |
| Supramolecular complex (GO:0099080) | 1 | 0.8 | Tubb3 |
| Synapse part (GO:0044456) | 4 | 3.3 | Syngr3, Slc32a1, Samd14, Slc18a3 |
| Synapse (GO:0045202) | 4 | 3.3 | Syngr3, Slc32a1, Samd14, Slc18a3 |

1. **Protein class**

|  | **#** | **%H** | **Gene** |
| --- | --- | --- | --- |
| Cell adhesion molecule (PC00069) | 1 | 2.9 | Thy1 |
| Chaperone (PC00072) | 1 | 2.9 | Clip3 |
| Cytoskeletal protein (PC00085) | 2 | 5.7 | Tubb3, Dmtn |
| Extracellular matrix protein (PC00102) | 2 | 5.7 | Hapln4, Spock2 |
| Gene-specific transcriptional regulator (PC00264) | 3 | 8.6 | Rbbp8, Crtc1, Per1 |
| Intercellular signal molecule (PC00207) | 3 | 8.6 | Vgf, Sst, Ccl9 |
| Membrane traffic protein (PC00150) | 4 | 11.4 | Dnajc6, Syngr3, Sncb, Syt3 |
| Metabolite interconversion enzyme (PC00262) | 7 | 20 | Plppr2, Trpt1, Mettl16, Pomgnt2, Pigz, Nat8l, Sgpp2 |
| Nucleic acid binding protein (PC00171) | 1 | 2.9 | Ncbp2 |
| Protein modifying enzyme (PC00260) | 5 | 14.3 | Masp1, Mest, Pcsk2, Adam8, Camkk1 |
| Protein-binding activity modulator (PC00095) | 1 | 2.9 | Rerg |
| Scaffold/adaptor protein (PC00226) | 2 | 5.7 | Btbd11, Ncdn |
| Transporter (PC00227) | 3 | 8.6 | Slc29a2, Ache, Slc18a3 |

Number of genes in each category (column #), the percentage of gene hits against the total number of genes (column %H) for treatment are indicated. GO: gene ontology; PC: protein class.

**Table S4.** Differential gene expression profile after Spinal Cord Injury (SCI) treated with polypyrrole/iodine plus swimming and enriched environment (PPy/I + SW/EE) vs SCI.

1. **Molecular Function**

|  | **#** | **%H** | **Gene** |
| --- | --- | --- | --- |
| Binding (GO:0005488) | 20 | 32.8 | Bcl2, Hbb, Spen, Dnah12, Cyr61, Stag1, Myrf, Parvg, Ccnl2, Kitlg, Dnah11, Vegfb, Tubb4a, Cntfr, Gpx3, Calb1, Junb, Erap1, Hba1, Cpne5 |
| Catalytic activity (GO:0003824) | 12 | 19.7 | Hbb, Dnah12, Plppr2, Ccnl2, Dnah11, Cdk1, Gpx3, Mgat3, Dpysl3, Dpysl5, Erap1, Hba1 |
| Molecular function regulator (GO:0098772) | 3 | 4.9 | Ccnl2, Kitlg, Vegfb |
| Molecular transducer activity (GO:0060089) | 8 | 13.1 | Olr768, Paqr4, Unc5b, Adgrl4, Ccnl2, Cdk1, Cntfr, Lpar4 |
| Structural molecule activity (GO:0005198) | 1 | 1.6 | Tubb4a |
| Transcription regulator activity (GO:0140110) | 3 | 4.9 | Myrf, Pou6f1, Junb |
| Transporter activity (GO:0005215) | 14 | 23 | Hbb, Slc9a1, Slc36a1, Slc16a3, Tth3, Kcng4, Slc35b3, Slc39a12, Slc17a5, Cacna1b, Kcnc3, Hba1, Slc18a3, Slco1c1 |

1. **Biological Process**

|  | **#** | **%H** | **Gene** |
| --- | --- | --- | --- |
| Biological adhesion (GO:002261) | 2 | 1.6 | Cyr61, Parvg |
| Biological regulation (GO:0065007) | 17 | 13.9 | Bcl2, Arhgap15, Cyr61, Slc9a1, Myrf, Olr768, Adgrl4, Ccnl2, Kitlg, Pou6f1, Vegfb, Slc39a12, Kank3, Calb1, Junb, Lpar4, Brinp1 |
| Cell population proliferation (GO:0008283) | 3 | 2.5 | Kitlg, Vegfb, Junb |
| Cellular component organization or biogenesis (GO:0071840) | 7 | 5.7 | Stag1, Unc5b, Parvg, Tubb4a, Dpysl3, Kank3, Brinp1 |
| Cellular process (GO:0009987) | 34 | 27.9 | Bcl2, Hbb, Spen, Dnah12, Cyr61, Slc9a1, Stag1, Myrf,  Olr768, Unc5b, Adgrl4,Parvg, Ccnl2, Gpcpd1, Kitlg, Dnah11, Pou6f1, Vegfb, Cdk1, Tubb4a, Slc39a12, Gpx3, Dpysl3, Dpysl5, Cacna1b, Kank3, Calb1, Junb, Erap1, Lpar4 |
| Developmental process (GO:0032502 | 7 | 5.7 | Unc5b, Parvg, Pou6f1, Vegfb, Hapln4, Junb, Brinp1 |
| Immune system process (GO:0002376) | 1 | 0.8 | Vegfb |
| Localization (GO:0051179) | 10 | 8.2 | Slc9a1, Slc36a1, Slc16a3, Kcng4, Vegfb, Slc39a12, Slc17a5, Cacna1b, Kcnc3, Slco1c1 |
| Locomotion (GO:0040011) | 2 | 1.6 | Unc5b, Vegfb |
| Metabolic process (GO:0008152) | 15 | 12.3 | Hbb, Spen, Myrf, Ccnl2, Gpcpd1, Pou6f1, Vegfb, Cdk1, Gpx3, Mgat3, Dpysl3, Dpysl5, Junb, Erap1, Hba1 |
| Multi-organism process (GO:0051704) | 1 | 0.8 | Junb |
| Multicellular organismal process (GO:0032501) | 4 | 3.3 | Unc5b, Vegfb, Hapln4, Brinp1 |
| Response to stimulus (GO:0050896) | 10 | 8.2 | Bcl2, Cyr61, Olr768, Unc5b, Adgrl4, Vegfb, Junb, Lpar4, Cpne5, Brinp1 |
| Signaling (GO:0023052) | 9 | 7.4 | Bcl2, Cyr61, Olr768, Adgrl4, Vegfb, Cacna1b, Calb1, Lpar4, Slc18a3 |

1. **Cellular component**

|  | **#** | **%H** | **Gene** |
| --- | --- | --- | --- |
| Cell junction (GO:0030054) | 1 | 0.6 | Parvg |
| Cell part (GO:0044464) | 34 | 21.4 | Bcl2, Arhgap15, Hbb, Spen, Dnah12, Slc9a1, Stag1, Myrf, Adgrl4, Parvg, Slc16a3, Ccnl2, Kitlg, Dnah11, Ttyh3, *Kcng4*, Pou6f1, Cdk1  Slc35b3, Tubb4a, Cntfr, Slc39a12, Slc17a5, Kank3, Kcnc3, Calb1, Junb, Erap1, Lpar4, Hba1 |
| Cell (GO:0005623) | 34 | 21.4 | Bcl2, Arhgap15, Hbb, Spen, Dnah12, Slc9a1, Stag1, Myrf, Adgrl4, Parvg, Slc16a3, Ccnl2, Kitlg, Dnah11, Ttyh3, Kcng4, Pou6f1, Cdk1  Slc35b3, Tubb4a, Cntfr, Slc39a12, Slc17a5, Kank3, Kcnc3, Calb1, Junb, Erap1, Lpar4, Hba1 |
| Extracellular region part (GO:0044421) | 6 | 3.8 | Hbb, Cyr61, Slc9a1, Vegfb, Gpx3, Hapln4, Hba1 |
| Extracellular region (GO:0005576) | 6 | 3.8 | Hbb, Cyr61, Slc9a1, Vegfb, Gpx3, Hapln4, Hba1 |
| Membrane part (GO:0044425) | 14 | 8.8 | Dnah12, Adgrl4, Slc16a3, Dnah11, Kcng4, Slc35b3, Cntfr, Slc39a12, Kcnc3, Calb1, Lpar4, Slc18a3, Slco1c1, Brinp1 |
| Membrane (GO:0016020) | 20 | 12.6 | Bcl2, Dnah12, Slc9a1, Adgrl4, Slc16a3, Kitlg, Dnah11, Ttyh3, Kcng4, Slc35b3, Cntfr, Slc39a12, Kcnc3, Calb1, Erap1, Lpar, Cpne5, Slc18a3, Slco1c1, Brinp1 |
| Membrane-enclosed lumen (GO:0031974) | 1 | 0.6 | Junb |
| Organelle part (GO:0044422) | 8 | 5 | Bcl2, Dnah12, Stag1, Dnah11, Slc35b3, Tubb4a, Junb, Slc18a3 |
| Organelle (GO:0043226) | 18 | 11.3 | Bcl2, Spen, Dnah12, Stag1, Myrf, Parvg, Ccnl2, Dnah11, Pou6f1, Cdk1, Slc35b3, Tubb4a, Slc17a5, Kank3, Calb1, Junb, Slc8a3, Brinp1 |
| Protein-containing complex (GO:0032991) | 10 | 6.3 | Hbb, Dnah12, Stag1, Dnah11, Kcng4, Cntfr, Kcnc3, Junb, Hba1, Slc18a3 |
| Supramolecular complex (GO:0099080) | 3 | 1.9 | Dnah11, Dnha12, Tubb4a |
| Synapse part (GO:0044456) | 2 | 1.3 | Calb1, Slc8a3 |
| Synapse (GO:0045202) | 2 | 1.3 | Calb1, Slc8a3 |

1. **Protein class**

|  | **#** | **%H** | **Gene** |
| --- | --- | --- | --- |
| Calcium-binding protein (PC00060 | 1 | 2.5 | Calb1 |
| Chaperone (PC00072) | 1 | 2.5 | Clip3 |
| chromatin/chromatin-binding, or -regulatory protein (PC00077 | 1 | 2.5 | Stag1 |
| Cytoskeletal protein (PC00085) | 6 | 15 | Dnah12, Parvg, Dnah11, Wasf1, Tubb4a, Kank3 |
| Extracellular matrix protein (PC00102) | 1 | 2.5 | Hapln4 |
| Gene-specific transcriptional regulator (PC00264 | 1 | 2.5 | Junb |
| Intercellular signal molecule (PC00207) | 3 | 7.5 | Cyr61, Vegfb, Cntfr |
| Metabolite interconversion enzyme (PC00262) | 8 | 20 | Plppr2, Pomgnt2, Gpcpd1, Gpx3, Mgat3, Dpysl3, Dpysl5, Lpcat4 |
| Nucleic acid binding protein (PC00171) | 1 | 2.5 | Spen |
| Protein modifying enzyme (PC00260) | 2 | 5 | Cdk1, Erap1 |
| Protein-binding activity modulator (PC00095 | 3 | 7.5 | Arhgap15, Rgs5 |
| Transmembrane signal receptor (PC00197) | 3 | 7.5 | Paqr4, Unc5b, Adgrl4 |
| Transporter (PC00227) | 9 | 22.5 | Slc9a1, Slc36a1, Slc16a3, Ttyh3, Slc35b3, Slc39a12, Scl17a5, Slc18a3, Slco1c1 |

1. **Pathway**

|  | **#** | **%H** | **Gene** |
| --- | --- | --- | --- |
| Apoptosis signaling pathway (P00006) | 1 | 3.2 | Bcl2 |
| Axon guidance mediated by netrin (P00009) | 1 | 3.2 | Unc5b |
| Axon guidance mediated by semaphorins (P00007) | 2 | 6.5 | Dpysl3  Dpysl5 |
| Blood coagulation (P00011) | 1 | 3.2 | Gp1bb |
| CCKR signaling map (P06959) | 1 | 3.2 | Bcl2 |
| Cytoskeletal regulation by Rho GTPase (P00016) | 1 | 3.2 | Tubb4a |
| Endogenous cannabinoid signaling (P05730) | 1 | 3.2 | Cacna1b |
| GABA-B receptor II signaling (P05731) | 1 | 3.2 | Cacna1b |
| Gonadotropin-releasing hormone receptor pathway (P06664) | 1 | 3.2 | Junb |
| Heterotrimeric G-protein signaling pathway-Gi alpha and Gs alpha mediated pathway (P00026) | 1 | 3.2 | Rgs5 |
| Heterotrimeric G-protein signaling pathway-Gq alpha and Go alpha mediated pathway (P00027) | 2 | 6.5 | Rgs5  Cacna1b |
| Huntington disease (P00029) | 1 | 3.2 | Tubb4a |
| Inflammation mediated by chemokine and cytokine signaling pathway (P00031) | 1 | 3.2 | Junb |
| Interleukin signaling pathway (P00036) | 1 | 3.2 | Cdkn1a |
| Ionotropic glutamate receptor pathway (P00037) | 1 | 3.2 | Cacna1b |
| Metabotropic glutamate receptor group II pathway (P00040) | 1 | 3.2 | Cacna1b |
| Metabotropic glutamate receptor group III pathway (P00039) | 1 | 3.2 | Cacna1b |
| Muscarinic acetylcholine receptor 1 and 3 signaling pathway (P00042) | 1 | 3.2 | Slc18a3 |
| Muscarinic acetylcholine receptor 2 and 4 signaling pathway (P00043) | 1 | 3.2 | Slc18a3 |
| Nicotinic acetylcholine receptor signaling pathway (P00044) | 1 | 3.2 | Slc18a3 |
| Oxidative stress response (P00046) | 1 | 3.2 | Bcl2 |
| PDGF signaling pathway (P00047) | 1 | 3.2 | Arhgap15 |
| Pyrimidine Metabolism (P02771) | 2 | 6.5 | Dpysl3  Dpysl5 |
| TGF-beta signaling pathway (P00052) | 1 | 3.2 | Junb |
| Thyrotropin-releasing hormone receptor signaling pathway (P04394) | 1 | 3.2 | Cacna1b |
| p53 pathway feedback loops 2 (P04398) | 1 | 3.2 | Cdkn1a |
| p53 pathway (P00059) | 2 | 6.5 | Cdkn1a |

Number of genes in each category (column #), the percentage of gene hits against the total number of genes (column %H) for treatment. GO, gene ontology. PC, protein class. P, pathway.

**Table S5.** DAVID functional annotation of gene up-regulated after spinal cord injury

(SCI) and treatment with plasma-synthesized polypyrrole/iodine (PPy/I).

| **Enrichment Score: 1.43** | **Count** | **P_Value** | **Benjamini** |
| --- | --- | --- | --- |
| [Methylation](http://www.uniprot.org/keywords/?query=Methylation) | 8 | 1.2E-3 | 4.6E-2 |
| [Synapse](http://www.uniprot.org/keywords/?query=Synapse) | 5 | 1.8E-2 | 4.0E-1 |
| [Cell junction](http://www.ebi.ac.uk/QuickGO/GTerm?id=GO:0030054) | 5 | 4.6E-2 | 3.8E-1 |
| [Cell junction](http://www.uniprot.org/keywords/?query=Cell%20junction) | 5 | 7.3E-2 | 6.7E-1 |
| [Cell membrane](http://www.uniprot.org/keywords/?query=Cell%20membrane) | 6 | 9.8E-1 | 1.0E0 |
| **Enrichment Score: 1.23** | **Count** | **P_Value** | **Benjamini** |
| Metal ion-binding site:Calcium 1; via carbonyl oxygen | 3 | 5.1E-3 | 6.0E-1 |
| Metal ion-binding site:Calcium 1 | 3 | 9.6E-3 | 5.8E-1 |
| Metal ion-binding site:Calcium 2 | 3 | 1.3E-2 | 5.4E-1 |
| [Calcium](http://www.uniprot.org/keywords/?query=Calcium) | 5 | 1.4E-1 | 7.9E-1 |
| [Metal ion binding](http://www.ebi.ac.uk/QuickGO/GTerm?id=GO:0046872) | 5 | 6.3E-1 | 1.0E0 |
| [Metal-binding](http://www.uniprot.org/keywords/?query=Metal-binding) | 7 | 7.3E-1 | 1.0E0 |
| **Enrichment Score: 0.98** | **Count** | **P_Value** | **Benjamini** |
| [Cleavage on pair of basic residues](http://www.uniprot.org/keywords/?query=Cleavage%20on%20pair%20of%20basic%20residues) | 4 | 2.2E-2 | 3.5E-1 |
| [Disulfide bond](http://www.uniprot.org/keywords/?query=Disulfide%20bond) | 13 | 6.2E-2 | 6.6E-1 |
| Signal peptide | 11 | 1.0E-1 | 9.1E-1 |
| [Secreted](http://www.uniprot.org/keywords/?query=Secreted) | 6 | 2.8E-1 | 9.0E-1 |
| Disulfide bond | 8 | 3.0E-1 | 1.0E0 |
| **Enrichment Score: 0.75** | **Count** | **P_Value** | **Benjamini** |
| [IGv](http://smart.embl.de/smart/do_annotation.pl?DOMAIN=SM00406) | 3 | 4.7E-2 | 8.5E-1 |
| [Immunoglobulin V-set](http://www.ebi.ac.uk/interpro/IEntry?ac=IPR013106) | 3 | 1.6E-1 | 1.0E0 |
| [Immunoglobulin-like fold](http://www.ebi.ac.uk/interpro/IEntry?ac=IPR013783) | 5 | 2.4E-1 | 1.0E0 |
| [Immunoglobulin-like domain](http://www.ebi.ac.uk/interpro/IEntry?ac=IPR007110) | 3 | 5.7E-1 | 1.0E0 |
| **Enrichment Score: 0.7** | **Count** | **P_Value** | **Benjamini** |
| [Endoplasmic reticulum membrane](http://www.ebi.ac.uk/QuickGO/GTerm?id=GO:0005789) | 5 | 1.1E-1 | 5.9E-1 |
| [Endoplasmic reticulum](http://www.uniprot.org/keywords/?query=Endoplasmic%20reticulum) | 5 | 1.6E-1 | 8.1E-1 |
| [Transferase](http://www.uniprot.org/keywords/?query=Transferase) | 6 | 4.6E-1 | 9.7E-1 |
| **Enrichment Score: 0.57** | **Count** | **P_Value** | **Benjamini** |
| [Integral component of membrane](http://www.ebi.ac.uk/QuickGO/GTerm?id=GO:0016021) | 23 | 2.3E-1 | 8.3E-1 |
| [Transmembrane](http://www.uniprot.org/keywords/?query=Transmembrane) | 25 | 2.5E-1 | 8.9E-1 |
| [Membrane](http://www.uniprot.org/keywords/?query=Membrane) | 28 | 3.0E-1 | 9.0E-1 |
| [Transmembrane helix](http://www.uniprot.org/keywords/?query=Transmembrane%20helix) | 24 | 3.2E-1 | 9.1E-1 |

The p-values associated with each terms inside the clusters is p-values by the Fisher

Exact Test which represents the “degree of enrichment” of the annotation term with the input

gene list. The Benjamini value is a parameter generated during adjustment of the P‑value

for multiple comparisons.

**Table S6.** DAVID functional annotation of the up-regulated gene expression after

spinal cord injury and application of polypyrrole/iodine plus swimming and enriched

environment (PPy/I+SW/EE).

| **Enrichment Score: 5.48** | **Count** | **P_Value** | **Benjamini** |
| --- | --- | --- | --- |
| [Hemoglobin complex](http://www.ebi.ac.uk/QuickGO/GTerm?id=GO:0005833) | 7 | 9.5E-12 | 1.1E-9 |
| [Oxygen transport](http://www.uniprot.org/keywords/?query=Oxygen%20transport) | 7 | 2.0E-11 | 2.4E-9 |
| [Globin](http://www.ebi.ac.uk/interpro/IEntry?ac=IPR000971) | 7 | 2.5E-11 | 4.9E-9 |
| [Globin-like](http://www.ebi.ac.uk/interpro/IEntry?ac=IPR009050) | 7 | 2.5E-11 | 4.9E-9 |
| [Globin, structural domain](http://www.ebi.ac.uk/interpro/IEntry?ac=IPR012292) | 7 | 3.8E-11 | 3.7E-9 |
| [Oxygen transport](http://www.ebi.ac.uk/QuickGO/GTerm?id=GO:0015671) | 7 | 4.4E-11 | 2.4E-8 |
| [Oxygen transporter activity](http://www.ebi.ac.uk/QuickGO/GTerm?id=GO:0005344) | 7 | 5.0E-11 | 8.3E-9 |
| Metal ion-binding site:Iron (heme distal ligand) | 6 | 5.0E-10 | 8.0E-8 |
| [Oxygen binding](http://www.ebi.ac.uk/QuickGO/GTerm?id=GO:0019825) | 7 | 1.5E-9 | 1.3E-7 |
| Metal ion-binding site:Iron (heme proximal ligand) | 6 | 3.1E-9 | 2.5E-7 |
| [African trypanosomiasis](https://david.ncifcrf.gov/kegg.jsp?path=rno05143$African%20trypanosomiasis&termId=550056431&source=kegg) | 7 | 1.6E-8 | 1.4E-6 |
| [Malaria](https://david.ncifcrf.gov/kegg.jsp?path=rno05144$Malaria&termId=550056432&source=kegg) | 7 | 2.5E-7 | 1.1E-5 |
| [Haemoglobin, beta](http://www.ebi.ac.uk/interpro/IEntry?ac=IPR002337) | 4 | 4.1E-6 | 2.6E-4 |
| [Heme](http://www.uniprot.org/keywords/?query=Heme) | 7 | 1.4E-5 | 5.6E-4 |
| [Hydrogen peroxide catabolic process](http://www.ebi.ac.uk/QuickGO/GTerm?id=GO:0042744) | 4 | 4.2E-5 | 1.1E-2 |
| [Haptoglobin binding](http://www.ebi.ac.uk/QuickGO/GTerm?id=GO:0031720) | 3 | 4.5E-5 | 2.5E-3 |
| [Heme binding](http://www.ebi.ac.uk/QuickGO/GTerm?id=GO:0020037) | 7 | 4.6E-5 | 1.9E-3 |
| [Iron](http://www.uniprot.org/keywords/?query=Iron) | 8 | 6.5E-5 | 1.9E-3 |
| [Haptoglobin-hemoglobin complex](http://www.ebi.ac.uk/QuickGO/GTerm?id=GO:0031838) | 3 | 7.9E-5 | 4.6E-3 |
| [Haemoglobin, pi](http://www.ebi.ac.uk/interpro/IEntry?ac=IPR002339) | 3 | 8.1E-5 | 3.9E-3 |
| Chain:Hemoglobin subunit beta-2 | 3 | 8.9E-5 | 4.7E-3 |
| [Iron ion binding](http://www.ebi.ac.uk/QuickGO/GTerm?id=GO:0005506) | 7 | 1.3E-4 | 4.5E-3 |
| [Haemoglobin, alpha](http://www.ebi.ac.uk/interpro/IEntry?ac=IPR002338) | 3 | 1.4E-4 | 5.2E-3 |
| [Response to hydrogen peroxide](http://www.ebi.ac.uk/QuickGO/GTerm?id=GO:0042542) | 5 | 2.2E-4 | 3.9E-2 |
| Sequence variant | 6 | 4.8E-4 | 1.9E-2 |
| [Positive regulation of cell death](http://www.ebi.ac.uk/QuickGO/GTerm?id=GO:0010942) | 4 | 1.2E-3 | 9.8E-2 |
| [Peroxidase activity](http://www.ebi.ac.uk/QuickGO/GTerm?id=GO:0004601) | 3 | 2.7E-3 | 7.3E-2 |
| [Erythrocyte development](http://www.ebi.ac.uk/QuickGO/GTerm?id=GO:0048821) | 3 | 3.8E-3 | 2.5E-1 |
| [Protein heterooligomerization](http://www.ebi.ac.uk/QuickGO/GTerm?id=GO:0051291) | 4 | 6.6E-3 | 3.6E-1 |
| [Blood microparticle](http://www.ebi.ac.uk/QuickGO/GTerm?id=GO:0072562) | 4 | 9.4E-3 | 3.0E-1 |
| [S-nitrosylation](http://www.uniprot.org/keywords/?query=S-nitrosylation) | 3 | 1.1E-2 | 1.6E-1 |
| [Polymorphism](http://www.uniprot.org/keywords/?query=Polymorphism) | 3 | 2.1E-2 | 2.2E-1 |
| [Metal-binding](http://www.uniprot.org/keywords/?query=Metal-binding) | 11 | 2.2E-1 | 7.2E-1 |
| [Acetylation](http://www.uniprot.org/keywords/?query=Acetylation) | 8 | 4.5E-1 | 9.2E-1 |
| **Enrichment score: 1.69** | **Count** | **P_Value** | **Benjamini** |
| [Response to corticosterone](http://www.ebi.ac.uk/QuickGO/GTerm?id=GO:0051412) | 4 | 5.9E-4 | 7.6E-2 |
| [Response to organic cyclic compound](http://www.ebi.ac.uk/QuickGO/GTerm?id=GO:0014070) | 7 | 6.0E-4 | 6.2E-2 |
| [Negative regulation of apoptotic process](http://www.ebi.ac.uk/QuickGO/GTerm?id=GO:0043066) | 7 | 1.4E-2 | 5.3E-1 |
| [Response to drug](http://www.ebi.ac.uk/QuickGO/GTerm?id=GO:0042493) | 7 | 1.5E-2 | 5.3E-1 |
| [Response to toxic substance](http://www.ebi.ac.uk/QuickGO/GTerm?id=GO:0009636) | 3 | 7.6E-2 | 9.3E-1 |
| [Regulation of cell cycle](http://www.ebi.ac.uk/QuickGO/GTerm?id=GO:0051726) | 3 | 7.8E-2 | 9.2E-1 |
| [Response to organic substance](http://www.ebi.ac.uk/QuickGO/GTerm?id=GO:0010033) | 3 | 1.1E-1 | 9.7E-1 |
| [Ubl conjugation](http://www.uniprot.org/keywords/?query=Ubl%20conjugation) | 4 | 5.4E-1 | 9.5E-1 |
| **Enrichment score: 1.3** | **Count** | **P_Value** | **Benjamini** |
| [Voltage-dependent potassium channel, four helix bundle domain](http://www.ebi.ac.uk/interpro/IEntry?ac=IPR027359) | 3 | 1.8E-2 | 3.9E-1 |
| [Ion transport](http://www.uniprot.org/keywords/?query=Ion%20transport) | 6 | 3.7E-2 | 3.4E-1 |
| [Regulation of ion transmembrane transport](http://www.ebi.ac.uk/QuickGO/GTerm?id=GO:0034765) | 3 | 5.6E-2 | 8.9E-1 |
| [Ion transport domain](http://www.ebi.ac.uk/interpro/IEntry?ac=IPR005821) | 3 | 6.1E-2 | 7.4E-1 |
| [Voltage-gated channel](http://www.uniprot.org/keywords/?query=Voltage-gated%20channel) | 3 | 7.7E-2 | 5.0E-1 |
| [Ion channel](http://www.uniprot.org/keywords/?query=Ion%20channel) | 4 | 8.9E-2 | 4.8E-1 |
| **Enrichment score: 1.14** | **Count** | **P_Value** | **Benjamini** |
| [Negative regulation of apoptotic process](http://www.ebi.ac.uk/QuickGO/GTerm?id=GO:0043066) | 7 | 1.4E-2 | 5.3E-1 |
| [PI3K-Akt signaling pathway](https://david.ncifcrf.gov/kegg.jsp?path=rno04151$PI3K-Akt%20signaling%20pathway&termId=550056323&source=kegg) | 5 | 7.1E-2 | 8.9E-1 |
| [Pathways in cancer](https://david.ncifcrf.gov/kegg.jsp?path=rno05200$Pathways%20in%20cancer&termId=550056444&source=kegg) | 5 | 1.1E-1 | 8.8E-1 |
| [Rap1 signaling pathway](https://david.ncifcrf.gov/kegg.jsp?path=rno04015$Rap1%20signaling%20pathway&termId=550056298&source=kegg) | 3 | 2.7E-1 | 9.7E-1 |
| **Enrichment score: 1.03** | **Count** | **P_Value** | **Benjamini** |
| [Endoplasmic reticulum](http://www.ebi.ac.uk/QuickGO/GTerm?id=GO:0005783) | 8 | 3.1E-2 | 4.5E-1 |
| [Endoplasmic reticulum membrane](http://www.ebi.ac.uk/QuickGO/GTerm?id=GO:0005789) | 5 | 1.5E-1 | 7.3E-1 |
| [Endoplasmic reticulum](http://www.uniprot.org/keywords/?query=Endoplasmic%20reticulum) | 5 | 1.8E-1 | 6.6E-1 |
| **Enrichment score: 0.84** | **Count** | **P_Value** | **Benjamini** |
| [Glycoprotein](http://www.uniprot.org/keywords/?query=Glycoprotein) | 16 | 7.1E-3 | 1.2E-1 |
| [Plasma membrane](http://www.ebi.ac.uk/QuickGO/GTerm?id=GO:0005886) | 22 | 4.3E-2 | 4.7E-1 |
| [Transmembrane helix](http://www.uniprot.org/keywords/?query=Transmembrane%20helix) | 28 | 1.3E-1 | 5.9E-1 |
| [Membrane](http://www.uniprot.org/keywords/?query=Membrane) | 32 | 1.3E-1 | 5.9E-1 |
| [Transmembrane](http://www.uniprot.org/keywords/?query=Transmembrane) | 28 | 1.3E-1 | 5.7E-1 |
| Glycosylation site:N-linked (glcnac...) | 15 | 1.4E-1 | 9.8E-1 |
| Transmembrane region | 16 | 1.9E-1 | 9.9E-1 |
| Topological domain:Cytoplasmic | 12 | 2.2E-1 | 9.9E-1 |
| [Integral component of membrane](http://www.ebi.ac.uk/QuickGO/GTerm?id=GO:0016021) | 23 | 4.4E-1 | 9.4E-1 |
| Topological domain:Extracellular | 8 | 5.0E-1 | 1.0E0 |
| [Cell membrane](http://www.uniprot.org/keywords/?query=Cell%20membrane) | 10 | 7.5E-1 | 9.9E-1 |
| **Enrichment score: 0.71** | **Count** | **P_Value** | **Benjamini** |
| [Disulfide bond](http://www.uniprot.org/keywords/?query=Disulfide%20bond) | 13 | 8.7E-2 | 5.0E-1 |
| [Signal](http://www.uniprot.org/keywords/?query=Signal) | 16 | 2.3E-1 | 7.1E-1 |
| Signal peptide | 11 | 3.7E-1 | 1.0E0 |
| **Enrichment score: 0.47** | **Count** | **P_Value** | **Benjamini** |
| [Immunoglobulin subtype 2](http://www.ebi.ac.uk/interpro/IEntry?ac=IPR003598) | 3 | 2.0E-1 | 9.9E-1 |
| [Igc2](http://smart.embl.de/smart/do_annotation.pl?DOMAIN=SM00408) | 3 | 2.1E-1 | 1.0E0 |
| [Immunoglobulin-like fold](http://www.ebi.ac.uk/interpro/IEntry?ac=IPR013783) | 5 | 2.9E-1 | 1.0E0 |
| [Immunoglobulin subtype](http://www.ebi.ac.uk/interpro/IEntry?ac=IPR003599) | 3 | 4.4E-1 | 1.0E0 |
| [Ig](http://smart.embl.de/smart/do_annotation.pl?DOMAIN=SM00409) | 3 | 4.6E-1 | 1.0E0 |
| [Immunoglobulin-like domain](http://www.ebi.ac.uk/interpro/IEntry?ac=IPR007110) | 3 | 6.2E-1 | 1.0E0 |
| **Enrichment score: 0.38** | **Count** | **P_Value** | **Benjamini** |
| [Extracellular region](http://www.ebi.ac.uk/QuickGO/GTerm?id=GO:0005576) | 5 | 2.7E-1 | 8.2E-1 |
| [Secreted](http://www.uniprot.org/keywords/?query=Secreted) | 5 | 5.1E-1 | 9.5E-1 |
| [Extracellular space](http://www.ebi.ac.uk/QuickGO/GTerm?id=GO:0005615) | 6 | 5.4E-1 | 9.6E-1 |
| **Enrichment score: 0.14** | **Count** | **P_Value** | **Benjamini** |
| [Transcription regulation](http://www.uniprot.org/keywords/?query=Transcription%20regulation) | 4 | 5.7E-1 | 9.6E-1 |
| [Transcription](http://www.uniprot.org/keywords/?query=Transcription) | 4 | 6.1E-1 | 9.7E-1 |
| [Regulation of transcription, DNA-templated](http://www.ebi.ac.uk/QuickGO/GTerm?id=GO:0006355) | 4 | 7.8E-1 | 1.0E0 |
| [Transcription, DNA-templated](http://www.ebi.ac.uk/QuickGO/GTerm?id=GO:0006351) | 3 | 8.1E-1 | 1.0E0 |
| [Nucleus](http://www.uniprot.org/keywords/?query=Nucleus) | 6 | 9.4E-1 | 1.0E0 |
| **Enrichment score: 0.1** | **Count** | **P_Value** | **Benjamini** |
| [Nucleotide-binding](http://www.uniprot.org/keywords/?query=Nucleotide-binding) | 5 | 7.0E-1 | 9.9E-1 |
| Nucleotide phosphate-binding region:ATP | 3 | 7.2E-1 | 1.0E0 |
| [ATP-binding](http://www.uniprot.org/keywords/?query=ATP-binding) | 3 | 8.9E-1 | 1.0E0 |
| [ATP binding](http://www.ebi.ac.uk/QuickGO/GTerm?id=GO:0005524) | 4 | 9.2E-1 | 1.0E0 |
| **Enrichment score: 0.01** | **Count** | **P_Value** | **Benjamini** |
| [Receptor](http://www.uniprot.org/keywords/?query=Receptor) | 7 | 9.0E-1 | 1.0E0 |
| [G-protein coupled receptor](http://www.uniprot.org/keywords/?query=G-protein%20coupled%20receptor) | 3 | 9.9E-1 | 1.0E0 |
| [Transducer](http://www.uniprot.org/keywords/?query=Transducer) | 3 | 9.9E-1 | 1.0E0 |
| [G-protein coupled receptor signaling pathway](http://www.ebi.ac.uk/QuickGO/GTerm?id=GO:0007186) | 3 | 1.0E0 | 1.0E0 |

The p-values associated with each terms inside the clusters is p-values by the Fisher

Exact Test which represents the “degree of enrichment” of the annotation term with

the input gene list. Benjamini FDR q-value is the correction for multiple comparison

implemented method by Benjamini in 1995.
